# A proinflammatory response and polarized differentiation of stromal elements characterizes the murine myeloma bone marrow niche

**DOI:** 10.1186/s40164-025-00606-x

**Published:** 2025-02-26

**Authors:** Hussein Ghamlouch, Dylan C. Gagler, Patrick Blaney, Eileen M. Boyle, Yubao Wang, Jason Avigan, Jinyoung Choi, Ola Landgren, Aristotelis Tsirigos, Francesco Maura, Gareth J. Morgan, Faith E. Davies

**Affiliations:** 1 Myeloma Research Program, Perlmutter Cancer Center, NYU Langone Health, 522 First Avenue, MSB4, New York, NY 10016 USA; 2 Applied Bioinformatics Laboratories (ABL), NYU Langone Medical Center, New York, NY USA; 3 Cancer Institute, University College London, London, UK; 4 Clinical Haematology Department, University College London Hospitals NHS Foundation Trust, London, UK; 5 Myeloma Division, Sylvester Comprehensive Cancer Center, University of Miami, Miami, FL USA

**Keywords:** Myeloma, Niche, Pro-inflammatory response, Bone marrow stress, Polarized differentiation, Mesenchymal stromal cells, Microenvironment, EndoMT

## Abstract

**Background:**

The bone marrow (BM) niche contains non-hematopoietic elements including mesenchymal stromal cells (MSC) and bone marrow endothelial cells (BMEC) which provide mechanical support, and control hematopoietic cell growth and differentiation. Although it is known that multiple myeloma (MM) cells interact closely with the BM microenvironment, little is known about the impact of MM on non-hematopoietic niche-forming cells.

**Methods:**

To address the role of the niche in MM pathogenesis, we utilized the 5TGM1 murine model. During the asymptomatic precursor stage of the model, we isolated the rare non-hematopoietic cells and performed single cell RNA sequencing. Using in-silico methods we characterized the individual cellular components of the niche, their relative abundance and differentiation state before and after exposure to MM cells as well as their intercellular interactions.

**Results:**

MM engraftment increased the abundance of MSC-lineage cells, BMECs and enhanced endothelial to mesenchymal transition. An inflammatory and oxidative stress signal was identified together with polarization of MSC differentiation away from osteocyte formation towards adipocytes which provide growth factors that are known to support MM expansion. BMEC differentiation was polarized towards sinusoidal endothelial cells with a pro-angiogenic/pro-inflammatory phenotype.

**Conclusions:**

MM cells impact the BM niche by generating a pro-inflammatory microenvironment with MSC differentiation being changed to generate cell subsets that favor MM growth and survival. In order to induce remission and improve long-term outcome for MM patients these inflammatory and oxidative stress signals need to be reduced and normal niche differentiation trajectories restored.

**Supplementary Information:**

The online version contains supplementary material available at 10.1186/s40164-025-00606-x.

## Background

The initiation and progression of multiple myeloma (MM) is characterized by the immortalization of a single clonal initiating cell, which migrates to the bone marrow (BM) where it binds to and modifies the stromal compartment to establish a MM niche. Acquired genetic variants in the MM cells drive the co-evolution of the clone within its supportive niche leading to progression from myeloma precursor disease states to MM [1–3]. Until recently, studies of progression have focused on the MM cell and studies of the tumor niche have focused on in-vitro culture analyses of specific cell populations. With increasing knowledge of the niche-forming stromal cells and access to single-cell analyses, it is possible to study the totality of the niche in-situ, and to understand its structure-function relationships.

The endosteal niche [4, 5] supports interactions between the MM clone and osteoclasts, with uncoupling of bone formation and resorption resulting in the development of characteristic lytic bone lesions. Much less is known about other non-hematopoietic BM elements including mesenchymal stromal cells (MSC) and bone marrow endothelial cells (BMEC). Importantly, interactions between the MM clone and non-hemopoietic cells have the potential to direct the niche to favor disease progression. Knowledge of BM niche has dramatically increased with the recent identification of multiple subpopulations of cells [6–11]. MSCs provide mechanical support and differentiate into osteoblasts, chondrocytes, and adipocytes, whereas BMECs differentiate into arterial and sinusoidal subtypes, regulating niche immune cell content, and neo-angiogenesis.

**Fig. 1:**
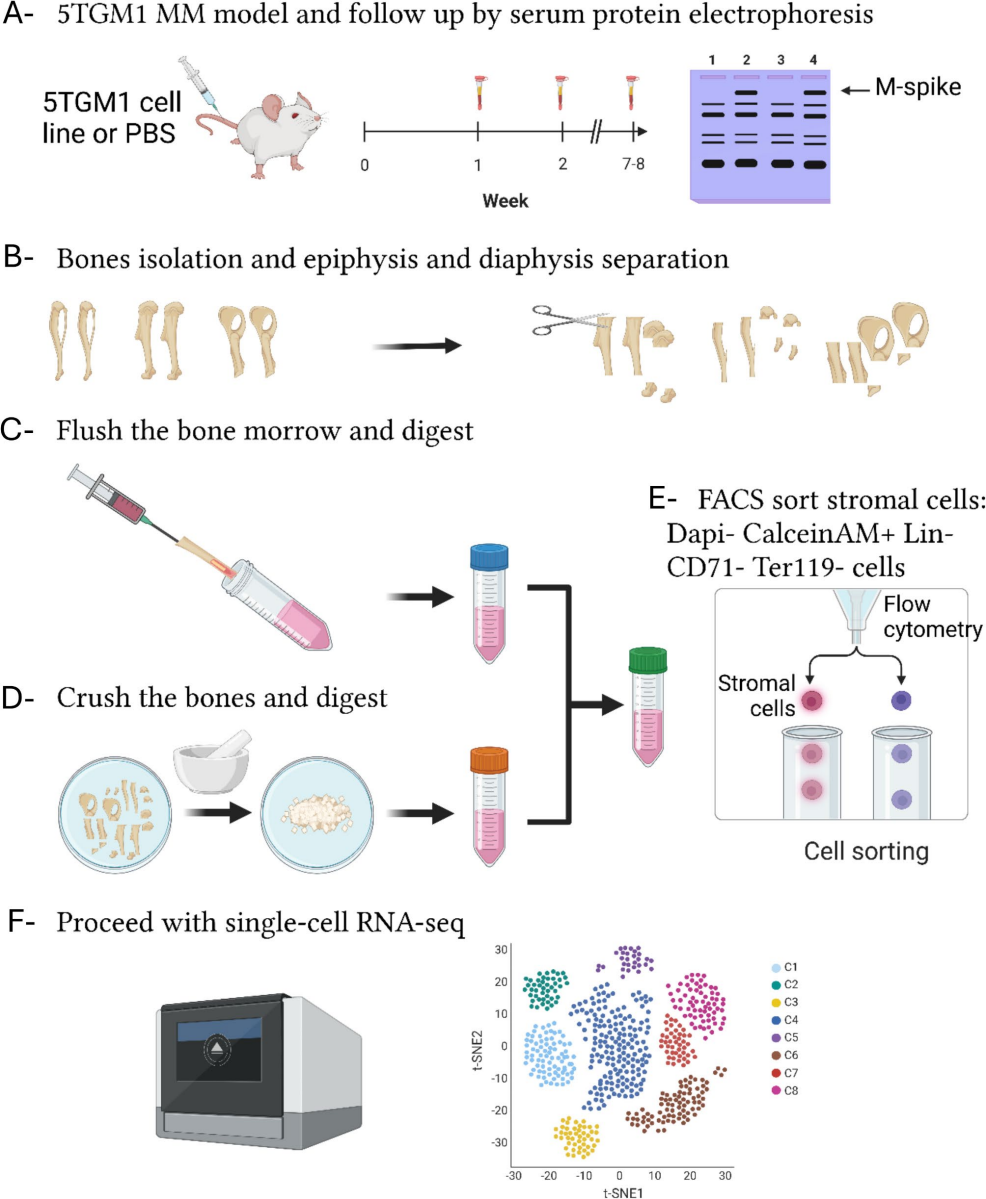
Overview of the study design. KaLwRij mice were injected with 0.5 × 10^6^ 5TGM1 cells or with PBS (**A**). Mice were bled weekly to check for the appearance of an M-spike in the serum by SPEP (**A**). Diseased and control mice were dissected, and tibia, femur and pelvic bones were isolated (**B**). BM cells were flushed and then digested by collagenase/dispase (**C**). Bones were crushed with a pestle and mortar and then subjected to digestion by collagenase/dispase (**D**). Digested BM and bones were mixed and stained for FACS sorting (**E**). BMSCs were enriched by sorting for live cells (7-AAD-/Calcein+) negative for erythroid (CD71/Ter119) and immune lineage markers (CD45/CD3/B220/CD19/Gr-1/CD11b) and 5TGM1 cells (CD45+). Cells were then subjected to the single-cell RNA sequencing pipeline (**F**). Figure created with BioRender.com

The long latency between MM initiation and clonal expansion suggests the niche needs to be substantially modified to permit MM growth. Analysis of the niche is complicated as the stromal components constitute only a small percentage of the total marrow content, are adherent, difficult to isolate and comprise multiple different sub-populations. By enriching for stromal cells using flow sorting combined with single-cell RNA sequencing (scRNA-seq), however, it is now possible to study these populations in murine models. The 5TGM1 murine model is an appropriate MM model for such studies [12–14] as it reflects human disease. Following BM engraftment of MM cells, we isolated non-hematopoietic elements and carried out scRNA-seq to capture the alterations of the stromal microenvironment during the asymptomatic phase of the disease (Fig. 1). We identified the individual cellular components of the BM niche, their relative abundance and differentiation state in-vivo before and after exposure to clonal plasma cells. We show that tumor engraftment increases abundance of MSC-lineage cells, BMECs and enhances endothelial to mesenchymal transition (EndoMT). The differentiation trajectories of MSCs were polarized away from osteocytes toward adipocytes providing growth factors further supporting MM clonal expansion and promoting inflammation, and neo-angiogenesis.

## Materials and methods

Here, we provide a brief description of the methods used; for more detailed information, please refer to the Supplementary Methods.

### The murine 5TGM1 model

**Fig. 2:**
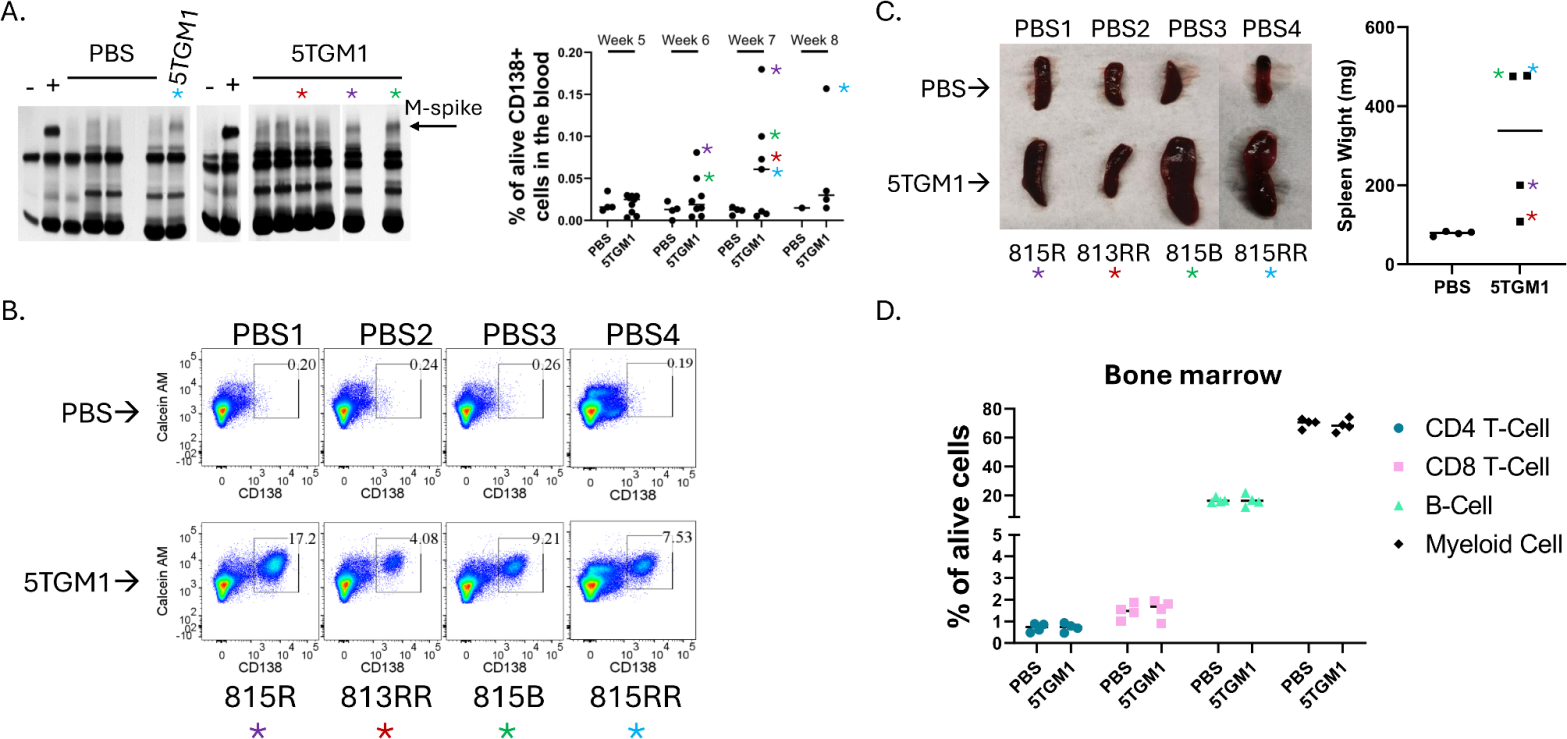
Characterization of the mice used for single cell analysis. (**A**) Left panel: Serum protein electrophoresis (SPEP) collected at 7 to 8 weeks. Right panel: Percentage of CD138 + cells in the peripheral blood. Asterisks indicate the 5TGM1 injected mice that were used for the single-cell RNA-seq analysis. (**B**) Flow cytometry plots showing the percentage of CD138 + 5TGM1 cells in the BM at 7–8 weeks after the injection. (**C**) Left panel: Image of spleens from PBS and 5TGM1-injected mice. Right Panel: Corresponding splenic weights. (**D**) Percentage of B-cells (B220+), CD4 + T-cell, CD8 + T-cell and myeloid cells (Gr1+/CD11+) in the BM of PBS and 5TGM1-injected mice

5TGM1 murine MM cells were obtained from Dr. Oyajobi. (University of Texas Health Science Center, TX). Female 25–28 week-old KaLwRij mice were injected intravenously with PBS alone (Control, healthy group) or with 0.5 × 10^6^ 5TGM1 cells (Myeloma group). Four control mice and 4 5TGM1-injected mice were selected for analysis based on the following criteria: (i) Presence of an M-spike with blood CD138 + cell frequency below 0.25% and with less than 20% infiltration of CD138 + 5TGM1 cells in the bone marrow (Fig. 2A, B, C), (ii) Mice were well-nourished, alert, and vivacious with no visible signs of symptomatic disease, (iii) Mice did not show any alterations in the hematopoietic compartment based on flow cytometry analysis (Fig. 2D). The mice were sacrificed at week 7–8 (Supplementary Table 1). The study was approved by the Institutional Animal Care and Use Committee at New York University Grossman School of Medicine (D16-00274 - PROTO201900102 - M4).

### Isolation and FACS enrichment of bone and bone marrow stroma cells

BM stroma cells were enriched by FACS sorting of live cells (Dapi-/Calcein+), which were negative for erythroid markers (CD71/Ter119) and immune lineage markers (CD45/CD3/B220/CD19/Gr1/CD11b) as previously described [15] (Supplementary Fig. S1).

### 10X single-cell RNA sequencing and library preparation

scRNA sequencing was performed according to the 10x Chromium (v3) protocol (Supplementary Methods). Resulting pooled libraries were sequenced on a Novaseq S1. Gene expression count matrices were generated using Cell Ranger v5.0, employing the refdata-gex-mm10-2020-A reference library provided by 10x Genomics.

### Single-cell RNA analysis

Following FACS sorting, mice were processed by condition (PBS or 5TGM1). In total, five libraries were created: two from 5TGM1-injected mice and three from PBS-injected mice. Cellranger outputs were processed in R using Seurat. Low quality cells with < 500 unique genes/transcripts, cells with > 5% mitochondrial transcripts and cells in the top 2% of unique genes and top 5% of unique transcripts were removed. The initial cell count was 52,911 and after the removal of low-quality cells, the final cell count was 45,030. These high-quality cells, distributed across 5 libraries, were integrated using the Seurat v3 framework [16]. For each library, the data was log-normalized by a scale-factor of 10,000, the top 2,000 variable features were identified, integration features were identified, the data was scaled, and underwent a principal component analysis with 50 PCs. Integration anchors were identified and the 5 libraries were integrated into a single Seurat object. The integrated dataset was scaled, dimensionality reduced with 30 PCs, finding neighbors, finding clusters with resolution 0.2, and UMAP generation. Cell type annotation was performed manually (Supplementary Fig. S2, S3, S4).

### Bootstrapping relative abundances

We utilized bootstrapping to statistically support the results. The cells from control PBS- and 5TGM1-injected mice were separated and randomly sampled from each subset with replacement 100 times and the relative abundances of each cell type per iteration was stored. Two-sample t-test was used to compare the mean relative abundances between conditions. Further analyses are described in detail in the supplementary methods.

## Results

### There is a differential abundance of stromal cell clusters between normal and MM

**Fig. 3:**
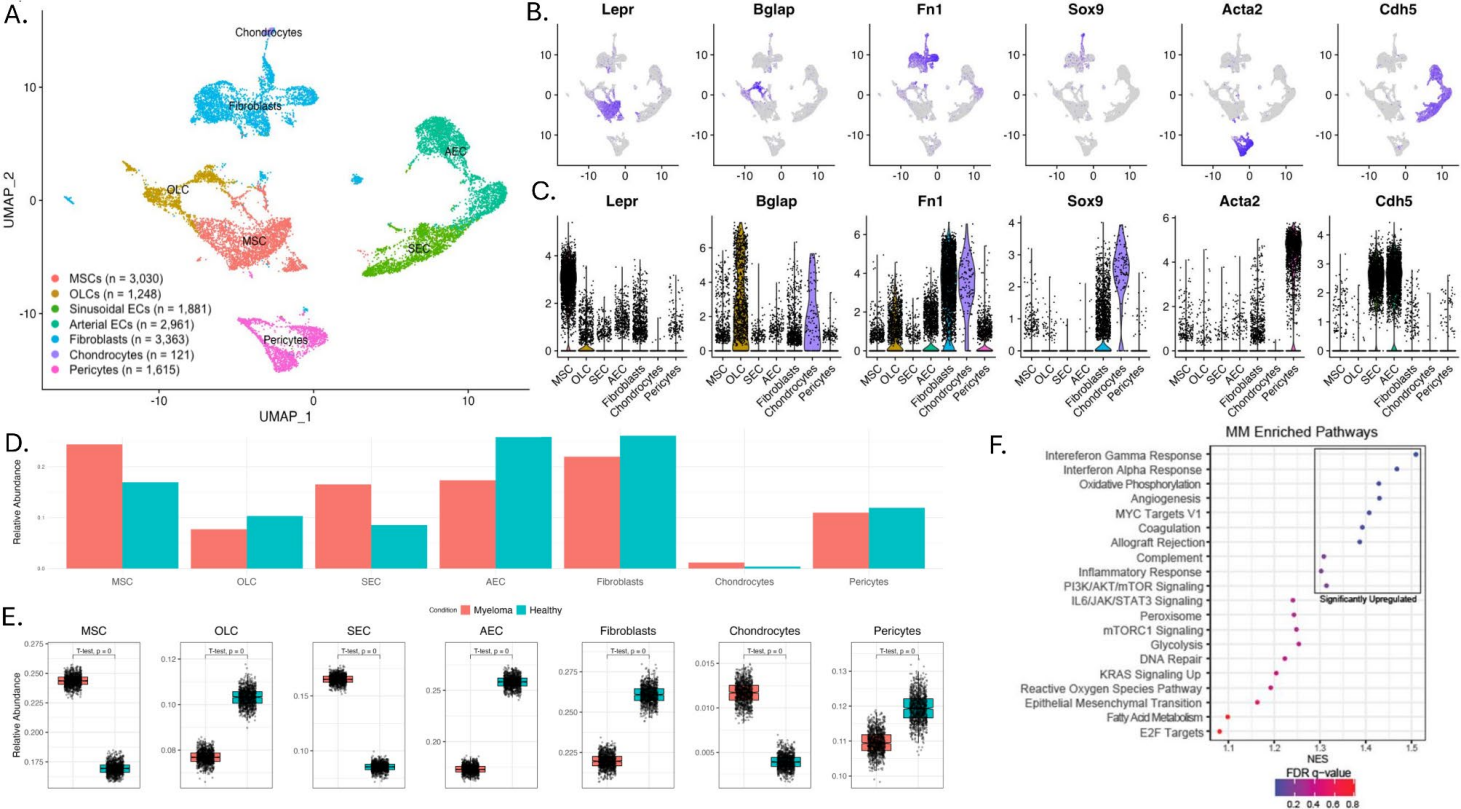
Overall results of single cell analysis of the stromal cells. (**A**) UMAP of the primary stromal populations. (**B**) UMAPs overlain with expression of stromal marker genes. (**C**) Violin plots showing expression of stromal marker genes across populations, (**D**) Barplots showing the relative abundances of stromal cell types between control and 5TGM1-injected mice (**E**) Boxplots showing bootstrapped (100x iterations) relative abundances of stromal populations between control and 5TGM1-injected mice. Whiskers represent the first and third quartiles and the ends of the whiskers represent the minimum and maximum values. Points beyond that are outliers. Notches in the boxplots represent the median value. *P*-values derived from t-test. *p* = 0 reflects *p*-value less than 2.225074e-308. (**F**) Dot plot showing the top 25 gene sets from pre-ranked GSEA between control and 5TGM1-injected mice, colored by FDR q-values. Pathways with FDR q-values < 0.25 considered significant

14,219 BM stromal cells were annotated based on their expression characteristics identifying 7 distinct populations including mesenchymal stem cells (MSCs) (*Lepr*, *Adipoq*, and *Cxcl12)*, osteo-lineage cells (OLCs) (*Bglap*, *Spp1*, and *Sp7)*, fibroblasts (*S100a4*, *Fn1*, and *Dcn)*, chondrocytes (*Col2a1*, *Sox9*, and *Acan)*, pericytes (*Acta2*, *Myh11*, and *Mcam)*, and endothelial cells (ECs) (*Cdh5*, *Cd34*, and *Pecam1)*, which were further annotated as either arterial endothelial cells (AEC) or sinusoidal endothelial cells (SEC) (*Flt4* aka VEGFR*-3*, *Ly6a* aka SCA*-1*, *Cxcl12*, and *Il6-st)* (Fig. 3A-C, Supplementary Fig. S2-S6).

The relative abundance of cell types between the two conditions differed. In the control mice, relative abundance was 16.9% MSCs, 10.3% OLCs, 8.5% SECs, 25.8% AECs, 26.1%, fibroblasts, 0.4% chondrocytes, and 11.9% pericytes, whereas in the 5TGM1-injected mice the relative abundance was, 24.4% MSCs, 7.7% OLCs, 16.5% SECs, 17.4% AECs, 22% fibroblasts, 1.2% chondrocytes, and 10.9% pericytes (Table 1; Fig. 3D and E). All differences in relative abundance between conditions were deemed significant by bootstrapping of means and two-sample *t*-tests. These data are consistent with MM engraftment being associated with an increase in the relative abundance of both MSCs and SECs.

**Table 1:** Absolute and relative abundances of stromal cell types and selected stromal subcluster populations

Cell Type		PBS Count	5TGM1 Count	PBS RA	5TGM1 RA	95% CI Lower	95% CI Upper	*p*-value
MSC		990	2040	0.169	0.244	0.073	0.076	1.69e-180
OLC		603	645	0.103	0.077	-0.027	-0.025	2.39e-122
SEC		499	1382	0.085	0.165	0.078	0.081	5.3e-198
AEC		1511	1450	0.258	0.174	-0.086	-0.083	9.26e-186
Fibroblasts		1525	1838	0.260	0.219	-0.043	-0.040	2.38e-115
Chondrocytes		23	98	0.004	0.012	0.008	0.008	8.16e-117
Pericytes		697	918	0.120	0.109	-0.012	-0.009	1.56e-37
Stromal Total		5848	8371					
MSC-lin Subcluster 0		607	1264	0.502	0.429	-0.076	-0.070	2.48e-97
MSC-lin Subcluster 1		169	464	0.185	0.119	-0.069	-0.064	2.02e-120
MSC-lin Subcluster 2		242	297	0.119	0.172	0.051	0.056	3e-97
MSC-lin Subcluster 3		246	186	0.075	0.174	0.097	0.102	4.27e-127
MSC-lin Subcluster 4		134	125	0.050	0.095	0.043	0.047	4.63e-93
MSC-lin Subcluster 5		14	172	0.069	0.011	-0.059	-0.057	1.66e-137
MSC-lin Total		1412	2508					
BMEC Subcluster 0		940	786	0.278	0.468	0.188	0.193	6.3e-183
BMEC Subcluster 1		276	1105	0.390	0.138	-0.254	-0.250	1.04e-233
BMEC Subcluster 2		508	455	0.161	0.252	0.089	0.093	1.54e-125
BMEC Subcluster 3		161	288	0.101	0.080	-0.022	-0.019	8.05e-67
BMEC Subcluster 4		122	167	0.059	0.060	0.000	0.002	0.0633
BMEC Subcluster 5		3	31	0.011	0.001	-0.010	-0.009	1.96e-79
BMEC Total		2010	2832					

Counts are absolute values of cells in each population and relative abundance (RA) values are mean values derived from our bootstrapping methodology. 95% confidence intervals refer to the difference in relative abundance between the compared groups. PBS – Phosphate Buffer Saline (Healthy Control); 5TGM1 – myeloma mouse model; RA – relative abundance; CI – confidence interval; MSC – mesenchymal stem cell; OLC – osteo-lineage cell; SEC – sinusoidal endothelial cell; AEC – arterial endothelial cell; MSC-lin – MSC-lineage subset comprised of MSC and OLC; BMEC – bone marrow endothelial cells comprised of SEC and AEC

### Inflammatory and oxidative stress signals are associated with MM

**Fig. 4:**
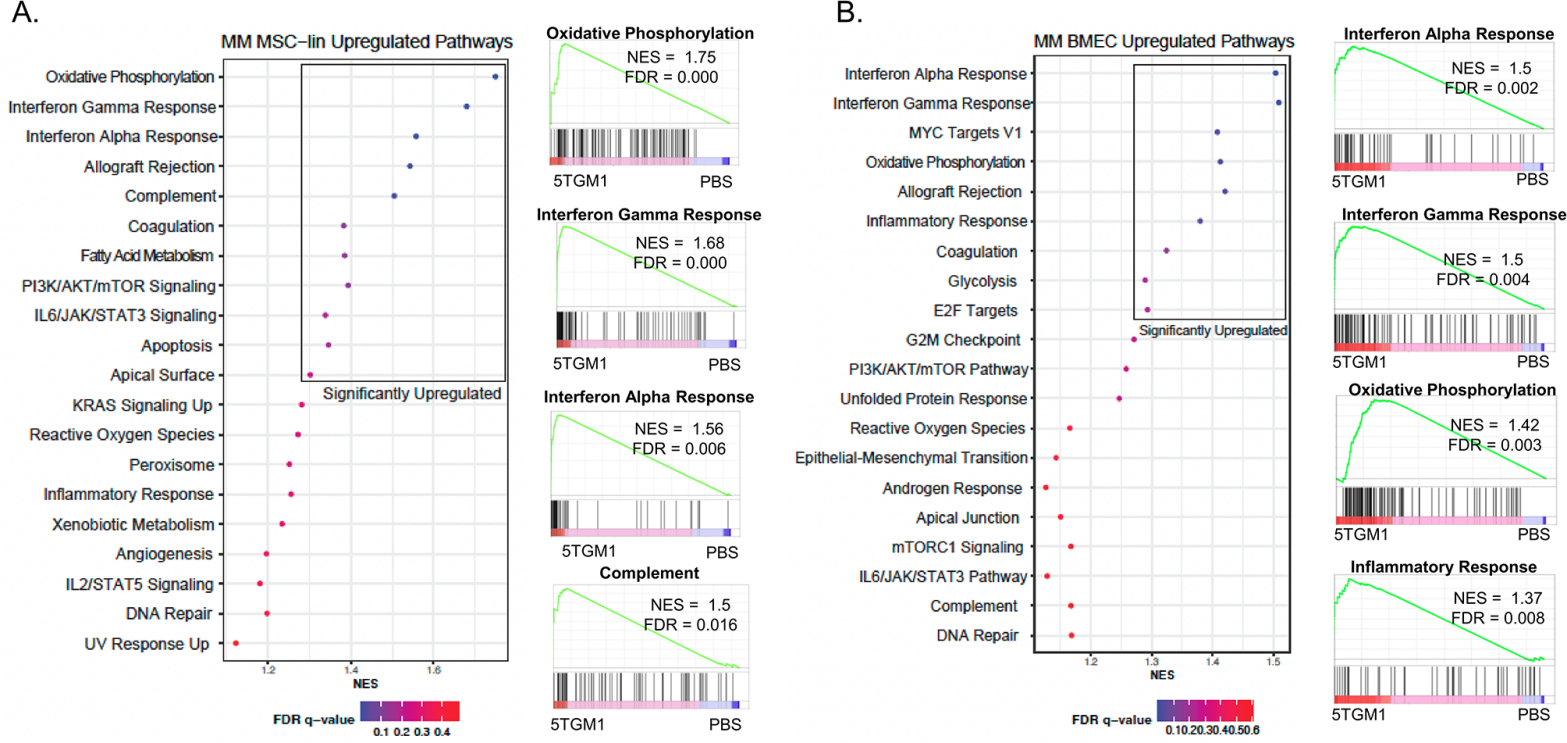
GSEA of pathways enriched in MSCs and BMECs from 5TGM1-injected mice. (**A**) Left panel: Dot plot showing the top 20 gene sets from pre-ranked GSEA between MSC-lineage subclusters control and 5TGM1-injected mice, colored by FDR. Right panel: Selected GSEA plots of pathways enriched in MM with FDR < 0.1. NES – normalized enrichment score; FDR – false discovery rate. (**B**) Left panel: Dot plot showing the top 20 gene sets from pre-ranked GSEA between BMEC subclusters in control and 5TGM1-injected mice, colored by FDR. Right panel: Selected GSEA plots of pathways enriched in MM with FDR < 0.1. NES – normalized enrichment score; FDR – false discovery rate

To elucidate the impact of the MM clone on the gene expression signatures seen in the stroma overall, as well as in the MSC and the BMEC populations specifically, we performed gene set enrichment analysis (GSEA). We used Preranked (v1) to broadly compare the control and 5TGM1-injected conditions using the collection of MSigDB hallmark gene sets. When looking at all of the stromal populations, 7 pathways were significantly enriched in 5TGM1-injected condition with FDR q-val < 0.1. These included interferon gamma response, interferon alpha response, angiogenesis, oxidative phosphorylation, MYC targets V1 and allograft rejection (Fig. 3F, Supplementary Table S2). For the MSCs, 5 gene sets were significantly enriched in 5TGM1-injected condition with an FDR q-val < 0.1, and these included oxidative phosphorylation, interferon-gamma response, interferon-alpha response, allograft rejection, and the complement pathway (Fig. 4A, Supplementary Table S3). For the BMECs, 6 gene sets were significantly enriched in 5TGM1-injected condition with an FDR q-val < 0.1, including interferon gamma response, interferon alpha response, allograft rejection, MYC targets V1, oxidative phosphorylation, and inflammatory response (Fig. 4B, Supplementary Table S4). Cumulatively, these results identify gene sets associated with a pro-inflammatory and oxidatively stressed microenvironment that is induced by the presence of clonal MM cells.

### MSC differentiation is polarized away from osteocytes towards adipocytes in MM

**Fig. 5:**
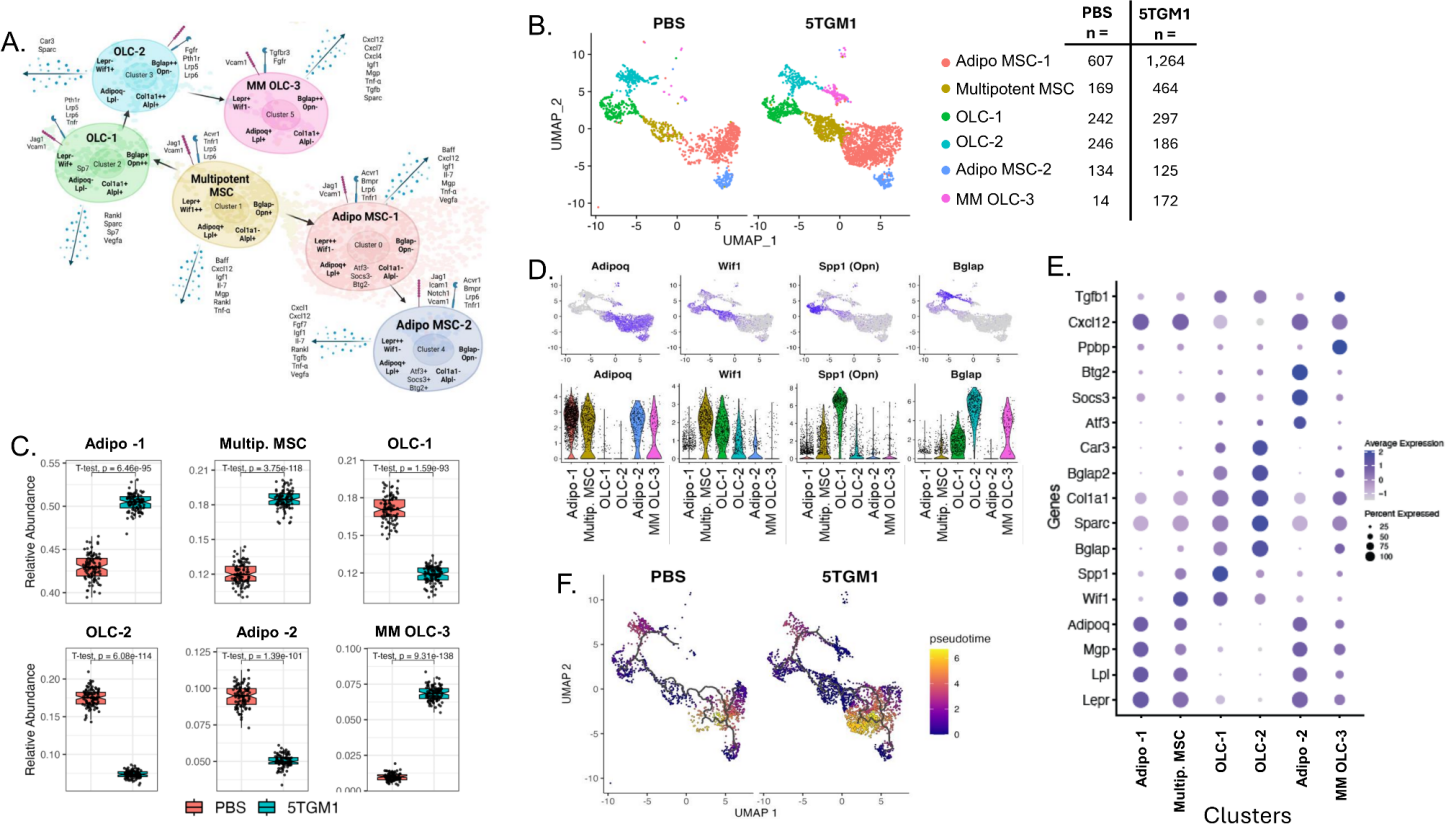
Mesenchymal stem cell lineage sub-clustering analysis. (**A**) Conceptual rendering showing the differentiation of the MSC-lineage subclusters and relevant expressed genes. Secreted molecules are represented by the blue dotted arrow, receptor molecules are represented by the blue surface molecule, and adhesion molecules are depicted by the maroon surface molecule. Bolded gene names inside the cell represent genes which distinguish clusters along the MSC differentiation trajectory. Unbolded gene names inside the cells represent intracellular molecules. Seurat cluster numbers are retained for clarity. (**B**) Condition split UMAP of the 6 MSC-lineage subclusters. (**C**) Boxplots showing mean relative abundances of bootstrapped (100x iterations) MSC-lineage subclusters between control and 5TGM1-injected mice. Whiskers represent the first and third quartiles and the ends of the whiskers represent the minimum and maximum values. Points beyond that are outliers. Notches in the boxplots represent the median value. *P*-values derived from two sample T-test. (**D**) Feature and violin plots showing expression of genes related to the differentiation of adipo-MSCs (*Adipoq*), multipotent MSCs (*Wif1*), early osteo-lineage cells (*Spp1* aka *Opn*), and mature osteoblasts (*Bglap*). (**E**) Condition split UMAP of the 6 MSC-lineage subclusters overlain with Monocle3 differentiation trajectories and calculated pseudotime values. (**F**) dot plot showing the expression of selected genes across all clusters

To better understand how MM may impact the differentiation and phenotypic status of the MSC-lineage cells, a sub-clustering analysis was performed of the combined MSC and OLC cells, as OLCs differentiate from MSCs. Six distinct subpopulations, were generated each of which had unique gene expression profiles (Fig. 5A-E, Supplementary Fig. S7A, S8, S9 and S10). Cluster 0 (which we named Adipo MSC-1) differentially expressed adipo-associated genes *Lepr*, *Lpl*, and *Mgp*, representing adipo-primed MSCs. Cluster 1 (MSC-0) highly expressed *Lepr*, *Lpl*, and *Mgp*, and differentially expressed *Wif1* (Wnt inhibitory factor 1), known to be associated with osteo-primed MSCs [17]. Cluster 2 (OLC-1) expressed *Wif1* but also expressed Osteopontin (*Spp1*) and moderate levels of *Bglap*, *Col1a1*, and *Sparc*, consistent with further osteo-lineage differentiation. Cluster 3 (OLC-2) expressed *Bglap*, *Bglap2*, and *Car3*, reflecting a further step towards a differentiated osteoblast. Cluster 4 (Adipo MSC-2) expressed moderate levels of *Lepr*, *Mgp*, and *Adipoq*, similar to Cluster 0, but also expressed several transcription factors including *Atf3*, *Socs3*, and *Btg2*. Cluster 5 (MM OLC-3) had an abnormal pattern, expressing genes associated with both an osteo and adipo-primed phenotype, including *Bglap*, *Col1a1*, *Lepr*, *Mgp*, and *Adipoq*, in addition to the chemokines *Cxcl7* and *Cxcl12*,* and Tgfb1.*

The relative abundance of the populations identified by the sub-clustering analysis varied between the control and 5TGM1 exposed marrow (Fig. 5C, Supplementary Fig S7A, Table 1). The MSC-0 and Adipo-MSC1 (clusters 0 and 1) were enriched in MM while both the early and late osteo-lineage cells, OLC-1 and OLC-2 (clusters 2 and 3) were decreased. Cluster 4 (Adipo MSC-2) was decreased in MM whereas cluster 5 (MM OLC-3) was found almost exclusively in MM. These results demonstrate that a key feature of MM development is a greater abundance of MSC-0 and adipo-primed MSCs in addition to fewer OLCs, consistent with the known decreased capacity for bone formation.

To further clarify the impact of MM on the differentiation trajectories of MSC cells we performed a trajectory analysis and computed pseudotime values for each cell using the Monocle3 R package (Fig. 5F and Supplementary Fig. S7B). We identified cluster MSC-0, the *Wif1* expressing, multi-potent *Lepr* + MSC as the origin of the differentiation process. We observed that pseudotime increased in 2 directions: towards adipo-primed MSCs and towards osteo-lineage cells, reflective of current understandings of MSC differentiation. Several of the trajectory routes that were identified were primarily populated by cell populations only seen in MM, specifically cells in cluster 0 adipo-MSCs and cluster 5 MM-OLCs. Taken together, these results imply that in the presence of MM cells, MSCs shift their differentiation trajectory away from osteocyte formation and towards adipocytes.

To investigate how the polarized MSC differentiation favored MM clonal expansion, we performed both an unsupervised and a supervised analysis of gene expression. For the unsupervised analysis, we performed a differential gene expression analysis in Seurat, comparing the control and 5TGM1 engrafted conditions. We observed upregulation of several genes relevant to MM biology in the latter condition, including *Vcam1*, *Jag1*, *Cxcr4*, *Igfbp3*, *Cxcl7*, *Plek*, and *Igf1* (Supplementary Fig. S8). Genes downregulated included *Runx2*, *Dock2*, *Ank3*, *Adamts9*, *Tmem86a*, *Greb1*, and *Tiparp* (Supplementary Fig. S8B). In a supervised analysis using a manually curated set of genes known to be important in MM growth and survival (Supplementary Fig. S8), we identified increased expression of molecules involved in the Notch pathway, and greater expression of *Vegf-a* in MSC-lineage clusters 0, 2 and 4 and *Igf1* in clusters 0, 1, and 4. We observed expression of Baff (*Tnfsf13b*), which provides a key survival signal for MM cells, only in clusters 0 and 1, populations that were both more abundant in MM (Supplementary Fig. S9,10). Taken together these analyses highlight the importance of uncoupling of bone resorption and formation, the pro-tumorigenic role of adipocytes, and the provision of MM growth and survival factors by MSCs within the niche.

### BMEC differentiation is polarized towards the generation of a pro-angiogenic and endoMT phenotype

**Fig. 6:**
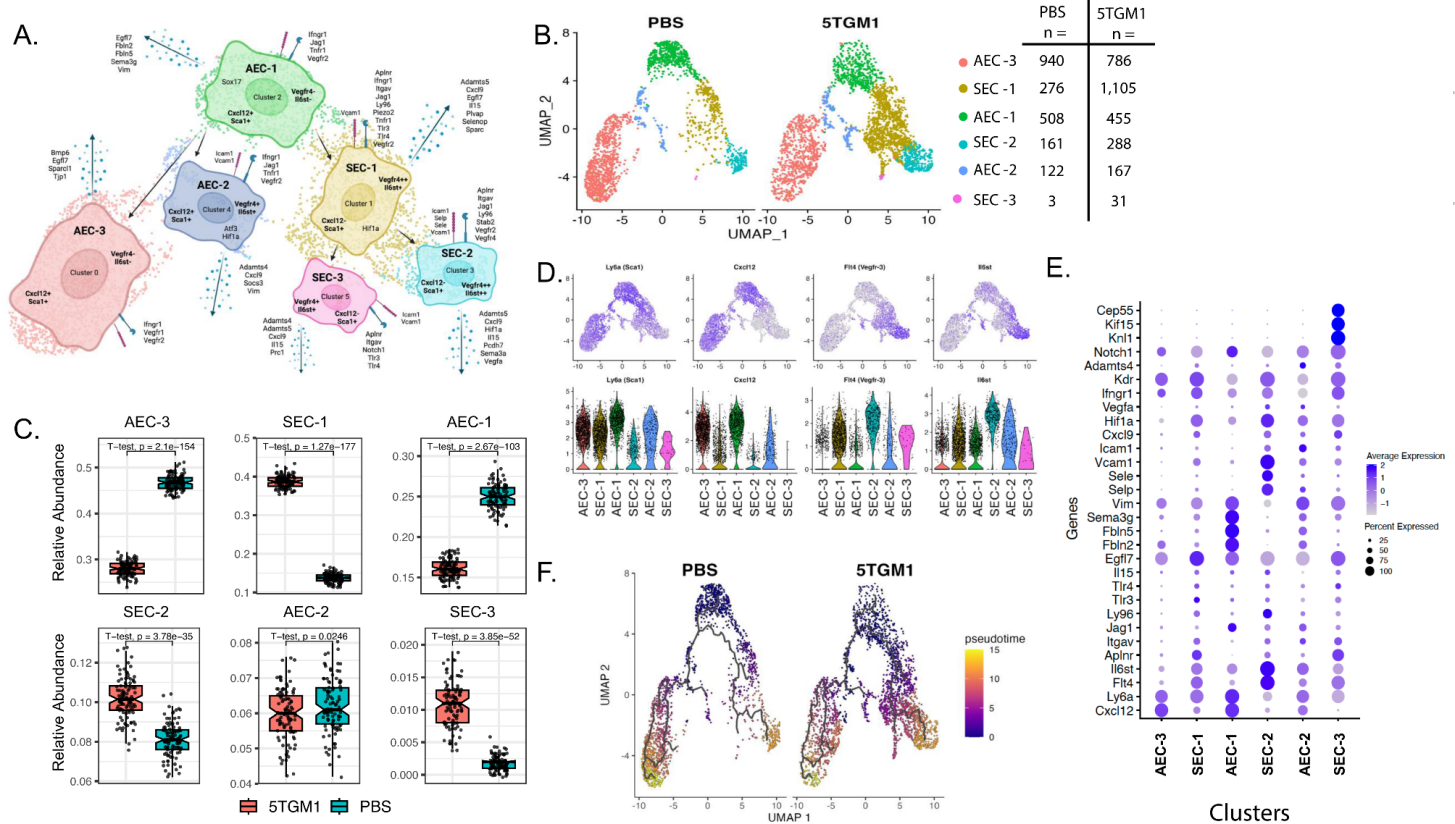
Bone marrow endothelial cell sub-clustering analysis. (**A**) Conceptual rendering showing the differentiation of the BMEC subclusters and relevant expressed genes. Secreted molecules are represented by the blue dotted arrow, receptor molecules are represented by the blue surface molecule, and adhesion molecules are depicted by the maroon surface molecule. Bolded gene names inside the cell represent genes which distinguish clusters along the MSC differentiation trajectory. Unbolded gene names inside the cells represent intracellular molecules. Seurat cluster numbers are retained for clarity. AEC – arterial endothelial cells; SEC – sinusoidal endothelial cells. (**B**) Condition split UMAP of the 6 BMEC subclusters. (**C**) Boxplots showing mean relative abundances of bootstrapped (100x iterations) BMEC subclusters between control and 5TGM1-injected mice. Whiskers represent the first and third quartiles and the ends of the whiskers represent the minimum and maximum values. Points beyond that are outliers. Notches in the boxplots represent the median value. *P*-values derived from two sample T-test. (**D**) Feature and violin plots showing expression of marker describing arterial (*Ly6a*high and *Cxcl12*high) and sinusoidal (*Flt4*high and *Il6st*high) endothelial cells. (**E**) dot plot showing the expression of selected genes across all clusters. (**F**) Condition split UMAP of the 6 BMEC subclusters overlain with Monocle3 differentiation trajectories and calculated pseudotime values

To study how MM may impact the differentiation and phenotype of BMECs, a sub-clustering analysis of combined AEC and SEC cells was performed. Six distinct subclusters were identified, each with unique gene expression profiles (Fig. 6A-Eand Supplementary Fig. S13, S14 and S15). Cluster 0 (AEC-3) a population of AECs expressed *Cxcl12*+/*Sca*1+ and lacked *Vegfr3*- and *Il6st*-. Cluster 1, (SEC-1), a SEC population expressed *Cxcl12*-/*Sca1* + and *Vegfr3*++/*Il6st*+. This cluster also expressed a variety of genes related to immune modulation and angiogenesis including *Aplnr*, *Itgav*, *Jag1*, *Ly96*, *Tlr3*, *Tlr4*, *Vegfr2*, and *Il15*. Cluster 2 (AEC-1) expressed *Cxcl12*+/*Sca1* + and *Vegfr3*-/*Il6st*- as well as *Egfl7*, *Fbln2*, *Fbln5*, *Sema3g*, and *Vim*. Cluster 3 (SEC-2) expressed several adhesion molecules including *Selp*, *Sele*, *Vcam1*, and *Icam1*, in addition to the receptor molecules *Aplnr*, *Itgav*, *Vegfr2*, and *Vegfr3* and the secreted molecules *Cxcl9*, *Hif1a*, *Il15*, and *Vegfa*. Cluster 4 (AEC-2) was positive for *Cxcl12*, *Sca1*, *Vegfr3*, *Il6st*, expressed *Icam1* and *Vcam1*, the receptor molecules *Ifngr1*, *Jag1*, and *Vegfr2*, and secreted *Adamts4*, *Cxcl9*, and *Vim*. Cluster 5 (SEC-3) is a small population that is *Cxcl12*-/*Sca1* + and *Vegfr*+/*Il6st*+, and expressed *Icam1* and *Vcam1*, the receptor molecules *Itgav*, *Notch1*, *Tlr3*, and secreted molecules *Adamts4*, *Cxcl9*, and *Il15*, in addition to several genes consistent with the cells being in cycle (*Knl1*, *Kif15*, *Cep55*).

These populations were seen at different relative abundances in the control and 5TGM1-injected mice (Fig. 6C, Supplementary Fig. S12A, Table 1). All 3 s populations were more abundant in the 5TGM1-injected condition, whereas AEC-1 and AEC-3 were decreased. These results demonstrate that a feature of MM during asymptomatic precursor stages is a greater abundance of sinusoidal ECs, consistent with their role in the mediation of the downstream immunologic composition of the marrow. To better understand the impact of BMECs we performed a pseudotime analysis of their differentiation trajectory and selected AEC cluster 1 as the root of the lineage with the trajectory running through to SECs (Fig. 6F Supplementary Fig. S12B).

To investigate how the polarized EC differentiation favored MM clonal expansion, we performed both an unsupervised and a supervised analysis of gene expression. The BMEC population had a greater expression of *Flna* and *Vim*, cytoskeleton proteins governing cell adhesion, in clusters 5 and 4, respectively. A greater level of expression of *Vegfr-3 (Flt4)*, *Itgav*, and *Aplnr* was seen in SEC clusters 1, 3, and 5; known to be associated with a pro-angiogenic phenotype [5] (Supplementary Fig. S13, S14). Increased expression of immune-regulating adhesion molecules *Selp*, *Sele*, *Vcam1*, and *Icam1* was seen in cluster 3 s, a key MM associated cluster (Supplementary Fig. S14). Furthermore, we identified increased expression of Toll-like Receptor genes *Tlr3* and *Tlr4*, also related to immune modulation, in SEC clusters 1 and 5 also a population almost unique to MM. Collectively, the results point to a BMEC response to MM whereby SEC cells become more abundant and have a pro-angiogenic and immune modulating phenotype. In a similar unsupervised analysis of genes upregulated in MM BMECs, we identified upregulation of *Cxcl4*,* Bst2*,* Stab2*,* Irf7*,* Igfbp4*, and *Adamts5*, among others (Supplementary Fig. S11). Genes depleted in MM included *Ebf1*,* Cdk8*,* Mecom*,* Epas1*, and *Syne1*, among others.

To explore the phenotypic potential of these differently abundant BMEC populations, we investigated differences in the gene expression patterns of relevant adhesion molecules, cytokines, chemokines, and soluble factors between MM and control. We noted increased expression of *Flna* and *Vim* in MM SEC clusters 1, 3, and 4 associated with a pro-angiogenic phenotype in MM associated endothelial cells (Supplementary Fig. S13-15). We identified genes enriched in MM including interferon induced proteins (e.g., *Ifi44*, *Ifit1)*, *Pf4* (Cxcl4), *Igfbp3*, and *Lgals1*. Genes decreased in MM included *Lifr*, *Cdkn1c*, *Pparg*, *Fbln2*, *Aqp1*, and *Tfpi*, (Supplementary Fig. S11).

Recent studies have demonstrated the plasticity of ECs in normal hematopoietic niches with the identification of a distinct population of ECs undergoing endothelial-mesenchymal transformation (EndoMT) which have the capacity to reconstitute the entire hematopoietic niche. Given that the niche in MM is hijacked and reprogrammed to support MM cell growth, we hypothesized that this rare cell type, co-expressing endothelial and stromal markers, would be increased in MM. A small population of cells (*n* = 381) with the EndoMT phenotype co-expressing both endothelial markers (*Cdh5*,* Pecam1*) and stromal markers (*Prrx1*,* Col1a1)* were identified (Supplementary Fig. S16). These cells also expressed transcription factors characteristic of EndoMT regulation (*Snai2*,* Twist1*, and *Zeb2*) and the HSPC mobilizing factor *Cxcl12*, suggesting that they are undergoing EndoMT [18]. Furthermore, upon GSEA of these cells compared to the remaining Cdh5 + cells we observed significant enrichment of the epithelial-mesenchymal transition pathway (NES = 1.218, FDR q-value = 0.1), (Supplementary Fig. S17). A slightly higher relative abundance of cells was also identified in MM (8.4% vs. 6.7%) providing further evidence for the complicated interplay between niche stromal cells, and their remodeling to support MM cell survival driven by inflammatory cytokines.

### Ligand–receptor interactions contribute to disease development and BM niche remodeling

In order to investigate the underlying signals that drive the molecular and cellular changes within the niche upon their interaction with MM cells, we used NicheNet, an algorithm which infers ligand-receptor interactions that trigger gene expression changes in cells engaged in the interactions [19]. By using transcriptomic data from interacting cells and merging it with known signaling and gene regulatory networks, it predicts the most impactful ligands produced by a sender cell, which subsequently influence gene expression in the recipient cell. We focused on identifying the ligands produced by MM cells during their interactions with stromal subpopulations. 5TGM1 cells were identified by their expression of characteristic of *Ighg2b* (IgG2b) within the ScRNA-seq data (Supplementary Fig. S17). We identified five active ligands (*Tgfb1*, *Pf4* (Cxcl4), *Cxcl12*, *Calr*, and *Sparc)*, expressed by MM cells (Supplementary Fig. S18-20, Supplementary Table S5). The stromal cell receptors that bind with these ligands were then identified. and showed multiple potential ligand-receptor interactions for each subset of niche cells. Interactions included: for MSCs, Tgfb1-Tgfbr3, Pf4-Fgfr2, and Calr-Lrp1; for OLCs, Tgfb1-Tgfbr3 and Sparc-Fgfr1; for fibroblasts, Tgfb1-Tgfbr3, Cxcl12-Ackr3, and Calr-Lrp1; for SECs, Tgfb1-Tgfbr2 and Sparc-Eng; and for AECs, Sparc-Eng. To gain deeper insights into the expression of *TGFB1* in human MM cells, we examined its expression across MM disease stages using Genomicscape [20]. *TGFB1* expression is increased in MGUS compared to normal plasma cells, and increases as the disease progresses (Supplementary Fig. S20C). This analysis highlights an intricate molecular web of communications between the MM cells and BM microenvironment and the pivotal role of Tgfb1 in niche remodeling.

## Discussion

We have characterized the non-hematopoietic stromal niche in normal and MM cell-exposed bone marrow, and identified seven major cellular subpopulations. Engraftment of the MM clone significantly altered their composition, differential abundance and expression patterns. Notably, we observed an increased relative abundance of MSC and BMEC subpopulations following engraftment. As our analysis was unrestricted to predefined stromal populations, we were able to identify significant skewing in MSC-lineage and BMEC differentiation trajectories, underscoring their contribution to niche modification favoring MM survival, clonal outgrowth, and development of the characteristic clinical features of MM.

Following MM engraftment, we observed a shift in MSC-lineage differentiation away from osteocyte towards adipocyte development. This shift was characterized by reduced osteocyte populations, along with an increased relative abundance and altered differentiation pattern toward adipocytes. Notably, the adipo-primed MSC population exhibited heightened expression of pro-MM growth and survival factors including vascular endothelial growth factor and insulin growth factor. Previous in-vitro studies have underscored the crucial bi-directional crosstalk between MM cells and adipocytes. Adipocytes play key roles in influencing normal bone formation, hematopoiesis, and in supporting MM cell clonal proliferation, migration, and homing [21]. For example adipocyte-derived *CXCL12* acts as a chemotactic factor for MM cells, and adipokines such as leptin promote MM proliferation and resistance to chemotherapy [22], stimulate osteoclastogenesis and inhibit osteoblastogenesis [23, 24], and act as an immune checkpoint [25]. In-vitro analyses using human MM cell lines and MSCs reveal that the exposure of pre-adipocytes to soluble MM-derived factors induces phenotypic changes. This exposure alters the differentiation pattern of adipocyte progenitors, leading to skewed metabolism-related transcripts and increased expression of inflammatory cytokines [26, 27]. In-vitro studies of MGUS/SMM MSCs also show a predisposition towards adipogenic differentiation, and suggest disease progression is supported through coordinated provision of fatty acid metabolites [28, 29]. However, there has been little work describing the differentiation trajectory of these populations in-vivo. The current study extends our understanding as pseudotime analysis shows that the relative expansion of adipo-primed MSC populations occurs at the expense of other MSC populations.

We identified a distinct, aberrant cellular population primarily present in MM, Cluster 5/MM OLC-3 (6.9% in MM, 1% in control), that expressed genes linked to both osteo and adipo-primed phenotypes. These cells produced cytokines and chemokines crucial for MM growth and survival. Further, we show that MSC-lineage cells exhibit an inflammatory, interferon and oxidative phosphorylation signature. This finding mirrors findings in other malignancies such as leukemia [30] where pro-inflammatory cytokines are potent inducers of malignant cell growth. Elevated oxidative phosphorylation induces adipogenesis and expands the MM niche through ROS-induced oxidative stress, promoting pro-MM growth factors like BAFF. The identification of pro-inflammatory signals in specific stromal populations is novel and aligns with previous bulk and single cell-sequencing of MM whole bone marrows and MSCs [31–33]. Mechanistically, it can be hypothesized that MM cells activate immune cells, inducing an inflammatory MSC phenotype, which leads to the production of inflammatory cytokines and DAMP-containing exosomes. Key pro-inflammatory cytokines derived from monocytes, NK cells, and CD8 + T cells, foster an inflammatory phenotype in stromal cells. In turn, inflammatory MSCs secrete factors supporting tumor cell proliferation and modulating the immune compartment, particularly impacting myeloid-derived cells [32].

Single-cell studies on MM patients highlight the crucial role of the immune microenvironment, particularly involving monocytes, NK, and T cells in disease progression [34]. Examining the role of the human niche in-vivo, however, is challenging as it requires analysis of both bone and bone marrow derived cellular populations. Available single cell human data suggests that cell types like MSCs are rare, limiting further characterization [15, 17, 29, 32, 34, 35]. Previously, Gooding et al. studied three murine specific MM endosteal niche populations including osteoprogenitors (Alcam + Sca1+), stromal progenitors (Sca1 + Alcam-), and endothelial cells (CD31+), and highlighted stromal precursor’ BMP signaling in MM survival [5]. By using unbiased selection of stromal populations, our results expand the understanding of the MM niche, identifying roles for pro-inflammatory signaling and polarized differentiation trajectories that support MM growth.

We observed a substantial increase in BMEC populations [8, 35, 36] which modulate innate immunity, mediate inflammation, and secrete MM proliferation factors. In MM, BMECs differentiate toward SECs, driving a pro-tumor response via altered cytokine signaling. This includes key cytokines like IL-1, IL-6, and IL-8, influencing neutrophil recruitment and immune cell trafficking. SECs are also known to exhibit immunosuppressive properties, stimulate neo-angiogenesis, and secrete pro-tumor factors. Our analysis demonstrates that both the MSC-lineage and BMECs exhibit a pro-inflammatory signature.

Importantly, our analysis of the interactions within the MM niche reveals the intricate dynamics between MM cells and various stromal cells and suggests a central role for TGFB1. TGFB1 influences cellular interactions, impacting adhesion, migration, proliferation, and extracellular matrix production [37, 38]. It also promotes osteoclast activity, contributing to bone breakdown, a hallmark of the disease [39–41]. This initiates a vicious cycle, releasing growth factors from the bone matrix, further fueling MM cell growth. TGFB1’s immunosuppressive effects allow MM cells to evade immune surveillance by inhibiting T cells and natural killer cells. Additionally, it induces EndoMT, a process in which ECs are driven towards a mesenchymal phenotype which is crucial in establishing the MM niche through stromal remodeling [42]. The translation of our findings regarding TGFB1 to human context was supported by a recent study that revealed a significant enrichment of TGFB1 signaling in MSCs derived from MGUS compared to those from healthy donors [29]. Additionally, MSCs from MGUS, SMM, and MM display a diminished capacity for osteogenic differentiation, likely driven by elevated TGFB1 signaling. Importantly, inhibiting TGFB1 signaling with an inhibitor restored both the normal MSC phenotype and their differentiation capacity [29].

### Limitations of the study

One notable limitation of this study is that it was conducted using a murine model. While the 5TGM1 model is well-established and recapitulates key aspects of human multiple myeloma, the findings may not fully translate to human disease due to species-specific differences. Conducting similar experiments using human-derived samples presents significant technical challenges, primarily due to the rarity of niche-forming stromal cells, which limits the ability to isolate and analyze these populations with the same resolution. Previous single-cell RNA-sequencing studies in humans have predominantly identified MSCs, with other stromal subtypes being too rare for comprehensive analysis.

Another limitation is the lack of validation of some of the RNA-seq findings at the protein level. Given the scarcity of the non-hematopoietic cells in the bone marrow, protein-level validation, such as through flow cytometry or immunohistochemistry, is difficult to perform robustly. While in vitro expansion of these rare cell populations could offer an alternative, this approach risks introducing culture-induced artifacts, potentially altering the expression patterns of key markers and confounding interpretation. Future studies will need to address these limitations by employing novel approaches to validate findings in human samples and at the protein level to strengthen the translational relevance of the results. Finally, although our single-cell transcriptomic analysis provides detailed insights into stromal cell dynamics, it does not directly measure absolute cell numbers, leaving open the possibility that observed changes in relative abundances could partly reflect shifts in other cell populations. Future studies incorporating spatial transcriptomics and proteomics analyses will be critical to validate these findings and clarify their implications.

Addressing these limitations in future research, particularly through the development of novel approaches for validating results in human samples and at the protein level, will enhance the translational relevance and impact of this work.

## Conclusions

**Fig. 7:**
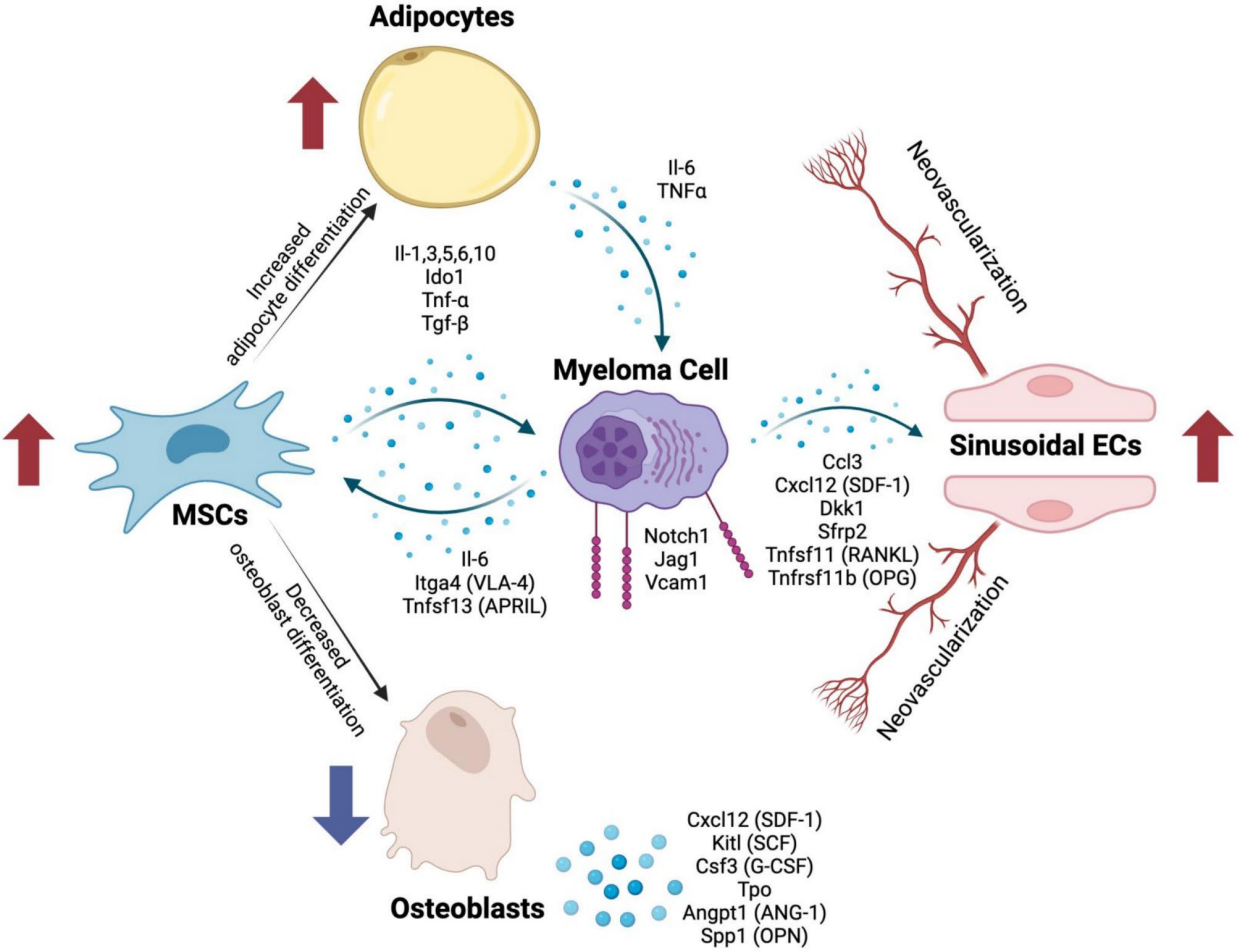
Conceptual overview of the interactions between myeloma cells and the stromal cell compartments. Secreted molecules are represented by the arrows with blue dots, and adhesion molecules are depicted by the maroon symbols on the myeloma cell. Cell types which increase in the context of MM are depicted by upward pointing red arrows and those that decrease are depicted by downward pointing blue arrows. Figure created with BioRender.com

In conclusion, our findings underscore the series of orchestrated interactions between stromal populations and the MM clone, leading to the creation of a pro-inflammatory microenvironment and polarized differentiation of niche components. This process leads to the expansion of niche populations that support clonal cells in the myeloma niche. Mechanistically, these changes notably shift MSC differentiation away from osteocytes towards adipocytes, and BMECs towards SECs, at the same time as increasing the proportion of EndoMT. Changes in niche cell populations might establish self-perpetuating loops that support clonal expansion. Therapeutically these loops will need to be broken to induce and maintain disease remission (Fig. 7).

## Electronic supplementary material

Below is the link to the electronic supplementary material.

Supplementary Material 1

Supplementary Material 2

Supplementary Material 3

## Data Availability

The sequence data will be publicly available in Gene Expression Omnibus (GEO). All other data are available from the corresponding authors upon request.

## Abbreviations

AEC: Arterial endothelial cells
BM: Bone marrow
BMEC: Bone marrow endothelial cells
ECs: Endothelial cells
EndoMT: Endothelial to mesenchymal transition
GSEA: Gene set enrichment analysis
MM: Multiple myeloma
MSC: Mesenchymal stromal cells
OLCs: Osteo-lineage cells
SECs: Sinusoidal endothelial cells
