# Supplementary material for: A proinflammatory response and polarized differentiation of stromal elements characterizes the murine myeloma bone marrow niche
Source: Exp Hematol Oncol. 2025 Feb 26;14:22. doi: 10.1186/s40164-025-00606-x (PMC11866767; doi:10.1186/s40164-025-00606-x)
Supplement: Supplementary file 1 — Supplementary Material 1 [file 40164_2025_606_MOESM1_ESM.pdf]

SUPPLEMENTARY METHODS

A pro-inflammatory response, bone marrow stress and polarised differentiation of stromal elements characterise the microenvironment of multiple myeloma.
Ghamlouch, Gagler, Blaney *et al*.

The mouse 5TGM1 model

The 5TGM1 murine MM cell line was obtained from Dr. Babatunde Oyajobi. (University of Texas Health Science Center at San Antonio, TX). This is a transplantable, non-radiation requiring model which exhibits the typical genetic features of human disease and reliably recapitulates the lytic bone disease typical of human MM (1, 2). Cells were grown in RPMI 1640 media supplemented with heat-inactivated 10% fetal bovine serum (FBS) and penicillin-streptomycin antibiotics and incubated at 37°C with 5% CO2. To comprehensively assess changes in the stromal cells during the asymptomatic stages of MM, 5TGM1 cells were intravenously injected into KaLwRij mice and stromal cells isolated following engraftment in the marrow. C57BL/KaLwRijHsd were purchased from Envigo (Horst, The Netherlands). Female age-matched 25-28-week-old mice were injected intravenously (tail vein) with 0.5×10^6 5TGM1 in 200 µL PBS (n=8) or with 200 µL PBS alone as a control group (n=4). A total of 12 mice were injected: 4 control mice with PBS and 8 mice with 5TGM1 cells. Based on pilot experiments, we established that a blood CD138+ 5TGM1 cell percentage between 0.05–0.25% correlates with less than 20% infiltration of CD138+ 5TGM1 cells in the bone marrow and a faint M-spike on SPEP analysis. We selected a total of 4 control mice and 4 5TGM1-injected mice for the analysis based on the following criteria:

1. Early disease stage: Presence of an M-spike with blood CD138+ cell frequency below 0.25% and less than 20% infiltration of CD138+ 5TGM1 cells in the bone marrow.
2. Healthy appearance: Mice were well-nourished, alert, and vivacious with no visible signs of symptomatic disease.
3. Hematopoietic stability: Mice did not show any alterations in the hematopoietic compartment based on flow cytometry analysis.

One of the 8 5TGM1-injected mice died at by the end of week 6 (day37 after injection) of unknown causes after biopsy. Three other mice were excluded from scRNAseq due to late disease onset. For the single-cell RNA sequencing and downstream analyses, 4 mice from each group (PBS-injected control and 5TGM1-injected) were selected to ensure robust statistical analysis and reproducibility. Three PBS-injected mice and three 5TGM1-injected mice were analyzed at week 7 (on day 48 after the 5TGM1 injection). One PBS-injected mouse and one 5TGM1-injected mouse were analyzed at week 8 (on day 55 after the 5TGM1 injection) (Supplementary Table1).

This study was approved by the Institutional Animal Care and Use Committee at New York University Grossman School of Medicine (D16-00274 - PROTO201900102 - M4).

Serum protein electrophoresis (SPEP) and detection of 5TGM1 by flow cytometry in the blood

SPEP was performed using the QuickGel Touch Split Beta SPE Kit (Helena Laboratories, Beaumont, Texas, USA), according to the manufacturer's instructions. Total white blood cells, were obtained from peripheral blood following ammonium chloride lysis of red blood cells. Cells were then stained with an anti-CD138-APC (Biolegend, Ref#142506, clone 281-2), anti-B220-PE (Biolegend, Ref#103208, clone RA3-6B2) and Dapi (Invitrogen, Ref#D1306) in PBS supplemented with 2% FBS (Corning, Ref# 35-010-CV). 5TGM1 cells were gated as alive Dapi- B220- CD138hi. Flow cytometry data were analysed using the FlowJo Version 10.8 software (TreeStar, Inc.).

Isolation of bone and bone marrow stroma cells

BM stromal cell isolation was performed according to Baryawno et al.(3) Mice were euthanised using CO_2 asphyxia followed by cervical dislocation. The femur, tibia and pelvic bones were harvested and placed in Media 199 (ThermoFisher Scientific, Ref#12350039) supplemented with 2% FBS (Corning, Ref# 35-010-CV). After muscle and tendon tissues were removed from the bones the diaphysis and epiphysis were separated. The BM was flushed from the diaphysis and was subjected to digestion with 1 mg/mL STEMxyme1 (Worthington, Ref#LS004106) and 1 mg/mL Dispase II (ThermoFisher Scientific, Ref#17105041), in Media 199 supplemented with 2% FBS for 25 min at 37°C in order to isolate the BM fraction of stromal cells. Stromal cells from the bone fraction were isolated by gently crushing and cutting bones (including epiphysis) into small fragments and digested in the same digestion mix as the bone marrow for 25 min, at 37°C with agitation (120 rpm). After digestion, both fractions were filtered through a 70µm filter (Fisher Scientific, Ref#08–771–2), pooled into one sample and red blood cell lysis was performed using ACK-lysis buffer (ThermoFisher Scientific, Ref#A1049201) for 5 minutes on ice. Cells were then stained in Media 199 supplemented with 2% FBS for FACS cell sorting**.**

FACS enrichment of bone and bone marrow stromal cells

Stromal cell enrichment was also performed as previously described (3). For flow cytometry and FACS, cells were resuspended in Media 199 supplemented with 2% FBS and stained for Ter119-APC (eBioscience, Ref#17–5921–82, clone TER–119), CD71-PECy7 (Biolegend, Ref#113812, clone RI7217), CD45-PE (eBioscience, Ref#12–0451–82, clone 30–F11), CD3-PE (Biolegend, Ref#100206, clone 17A2), B220-PE (Biolegend, Ref#103208, clone RA3–6B2), CD19-PE (Biolegend, Ref#115508, clone 6D5), Gr1-PE (Biolegend, Ref#108408, clone RB6–8C5), and CD11b-PE (Biolegend, Ref#101208, clone M1/70) for 30 minutes on ice. Dead cells and debris were excluded by FSC, SSC, Dapi (Invitrogen, Ref#D1306) and Calcein AM (ThermoFisher Scientific, Ref#C3099) profiles. FACS was performed on a BD FACSAria II sorter, and stromal cells were collected in Media 199 supplemented with 2% FBS and 0.4% UltraPure BSA (ThermoFisher Scientific, Ref#AM2616). Bone marrow stroma was enriched by sorting of live cells (Dapi-/Calcein+) negative for erythroid (CD71/Ter119) and immune lineage markers (CD45/CD3/B220/CD19/Gr1/CD11b) (Supplementary Fig. S2).

Flow cytometry analysis of CD138+ 5TGM1 cells and immune cell populations was performed before the enzymatic digestion in the bone marrow fraction only. Cells were

stained with CD4-APC (Invitrogen, Ref#MCD0405, clone RM4-5), CD8-BB515 (BD Biosciences, Ref#564422, clone 53-6.7), Gr1-PE (Biolegend, Ref#108408, clone RB6–8C5), and CD11b-PE (Biolegend, Ref#101208, clone M1/70), CD19-APC-Cy7 (Biolegend, Ref#115530, clone 6D5), CD138-BV605 (Biolegend, Ref#142516, clone 281-2) and B220-PercP-Cy5.5 (Biolegend, Ref# 103236, clone RA3-6B2).

Single-cell RNA analysis

Three PBS-injected and three 5TGM1-injected mice were analyzed on day 48 after injection (week 7), and one mouse from each group was analyzed on day 55 after injection (week 8). We initially intended to identify individual mice using a hashtaging approach with anti-mouse Hashtag-oligo antibodies (Biolegend, TotalSeq™-B301-308 anti-mouse), targeting CD45 and MHC I. However, this failed due to absent CD45 expression on stromal cells and low MHC I levels. For the three PBS-injected and three 5TGM1-injected mice analyzed on day 48 after injection (week 7), FACS-sorted cells from each mouse were pooled by condition (PBS or 5TGM1). In total, five libraries were created: two from 5TGM1-injected mice and three from PBS-injected mice. These were integrated using the Seurat v3 framework. Specifically, Cellranger outputs were processed in R using Seurat. In order to exclude low quality cells and multiplets or doublets, the percentage of mitochondrial transcripts, the number of unique genes per cell, and the number of unique transcripts per cell was computed. Specifically, cells with fewer than 500 unique genes or transcripts were removed in addition to those with greater than 5% mitochondrial transcripts and cells within the top 2% of unique genes and top 5% of unique transcripts. The initial cell count was 52,911 and after the removal of low-quality cells, the final cell count was 45,030, reflecting a 14.9% loss. Although the Hashtag-oligo data was normalized using a centered log-ratio (CLR) transform and demultiplexed using Seurat's HTODemux() function with kmeans and a 0.999 quantile cutoff, the data were not used further due to the lack of robust distinction between singlets and doublets/negatives. Instead, the 45,030 high quality cells were then integrated using the Seurat v3 framework. For each library, the data was log-normalised by a scale-factor of 10,000, the top 2,000 variable features were identified using the vst selection method, integration features were identified, the data was scaled, and underwent a principal component analysis with 50 PCs. Afterwards, integration anchors were identified using the FindIntegrationAnchors() function with a canonical correlation analysis and the 5 libraries were integrated into a single Seurat object using the IntegrateData() function. The integrated dataset was then run through a further Seurat workflow which involved scaling the data, dimensionality reduction with 30 PCs, finding neighbors, finding clusters with resolution 0.2, and UMAP generation. Cell type annotation was performed manually, using established markers of both the stromal and immune compartment (Supplementary Fig. S4, S5). After manual annotation of the quality filtered cells, it was observed that a majority of the cells (30,165 cells, 67% of total) were not stromal cells and in fact were a combination of erythrocytes, leukocytes, granulocytes, and progenitor cells (Supplementary Fig. S2 and Supplementary Table 6). These non-stromal cells were removed from downstream analysis, the remainder were reclustered and a further 646 cells identified as additional leukocytes were removed, leaving 14,219 high-quality stromal cells comprised by MSCs, OLCs, sinusoidal EC, arterial EC, fibroblasts, chondrocytes, and pericytes.

Bootstrapping Relative Abundances

In the absence of successful hashtag sequencing results, we utilized a bootstrapping methodology to statistically assess differences in cell populations between conditions. To that end, we separated the cells from PBS and 5TGM1-injected conditions and randomly sampled from each subset with replacement 100 times and stored the relative abundances of each cell type per iteration. We then performed a two-sample t-test to compare the mean relative abundances between conditions. The same methodology was used to compare relative abundances between conditions for the MSC-lineage and BMEC subsets.

Sub-clustering Analysis

Sub-clustering analyses were performed on the cell populations of interest using a standard Seurat workflow, which included scaling the data, finding highly variable features, a dimensionality reduction (10 PCs for MSC-lineages and 7 PCs for BMEC), finding nearest neighbors, and clustering with different resolutions (0.2 for MSC-lineage and 0.15 for BMECs). After removing 4 small subclusters with leukocyte expression from the MSC-lineage, comprising 358 cells, this yielded 6 subclusters for both the MSC-lineage cells (n = 3,920) and BMEC cells (n = 4,842). The identification of 5TGM1 cells was based on the expression of Ighg2b in the full dataset prior to subclustering. After identification, these cells were subclustered with stromal cells using the standard Seurat workflow. This workflow included data scaling, identification of highly variable features, dimensionality reduction (30 PCs), nearest-neighbor identification, and clustering with a resolution of 0.05.

Heatmaps and GSEA analysis

To explore differences in cellular phenotype, differential gene expression analyses were performed using Seurat's FindAllMarkers() and FindMarkers() functions to identify genes differentially expressed between subclusters and between the PBS- and 5TGM1-injected conditions, respectively. The most significant differentially expressed genes by adjusted p-value were used for generating heatmaps of scaled expression using the ComplexHeatmap package. GSEAPreranked v1 was used to identify gene sets which were differentially enriched between the PBS and 5TGM1-injected conditions. Ranked lists were generated by finding differentially expressed genes in each condition for the cell types of interest and multiplying their average log2 fold-change by the negative log of their adjusted p-value.

Pseudotime/trajectory analysis

The Monocle3 R software package1 was used to construct single-cell trajectories and compute pseudotime for the MSC-lineage populations and the BMEC populations, respectively. The workflow followed a standard Monocle workflow which involved converting Seurat objects of our cell populations of interest into Monocle objects using as.cell_data_set() function, learning the sequence of gene expression changes each cell goes through as a part of a dynamic process to place cells within an overall trajectory using the learn_graph() function, and computing pseudotime, which is a measure of how much progress an individual cell has made through the computed trajectory. Roots for computing pseudotime (i.e. pseudotime = 0) were selected as the cells within subcluster

1 (Multipotent MSC) for the MSC-lineage cells and subcluster 2 (AEC-1) for the BMECs. For more methodological details see the Monocle3 pipeline https://cole-trapnell-lab.github.io/monocle3/docs/trajectories/.

EndoMT analysis

In order to identify cells undergoing endothelial-mesenchymal transformation (EndoMT), we pursued an analytical isolation strategy adapted from Kenswil et al.(4)Our adaptation of the workflow was as follows: we subset out BMEC and MSC-lin cells from our full stromal cell object and ran a standard workflow on the extracted cells, using 10 PCs and resolution = 0.2 for clustering. Although most Cdh5+ cells were found in the BMEC cluster, some of these cells were found in the MSC-lin cluster. Conversely, some cells found within the BMEC cluster expressed MSC-lin genes such as Col1a1 and Prrx1, suggesting that there may be a transitional population of cells which expresses both BMEC and MSC-lin genes. As such, we extracted all of the cells from our BMEC/MSC-lin subset that had positive expression of *Cdh5* and ran another standard workflow on that object, again with 10 PCs and resolution = 0.2.

After visually inspecting the expression of genes associated with EndoMT including *Cdh5*, *Pecam1*, *Emcn*, *Prrx1*, *Col1a1*, *Cxcl12, Snai2*, *Twist1*, *Zeb2*, and *Yap1*, we identified 2 putative clusters of cells that matched the EndoMT profile and clustered away from the remaining Cdh5 positive cells (clusters 4, 6, and 8). In particular, these clusters showed decreased expression of BMEC markers (*Cdh5, Pecam1, Emcn*), increased expression of MSC-lin markers (*Prrx1, Col1a1, Cxcl12*), and expression of transcription factors involved in EndoMT (*Twist1, Snai2, Zeb2, Yap1*), indicative of cells undergoing EndoMT(5). Furthermore, upon gene set enrichment analysis (GSEA) of these cells compared to the remaining Cdh5+ positive cells, we observed significant enrichment of the Epithelial-Mesenchymal Transition pathway (NES = 1.218, FDR q-value = 0.1).

Cell communication analysis using NicheNet

Ligand-receptor interactions were established using the ligand-receptor interaction database from the Nichenetr R package (6). The Nichenetr algorithm prioritises ligands expressed by "sender" cells according to their capacity to activate a specific set of target genes in "receiver" cells. Differential gene expression (with log2FC > 0.25 and adjusted P value < 0.05) was assessed in 5TGM1-injected vs. control conditions for each stromal cell subset, guiding the selection of the target gene set in the receiver cells. Nichenetr's default approach was adopted for specifying background gene expression. Genes were considered expressed if present in at least 10% of cells within a cell type. For each “receiver” cell type (i.e., MSCs, Fibroblasts, Arterial ECs, Pericytes, Sinusoidal ECs and OLCs), we performed the analysis separately and for each analysis the 5TGM1 cells cluster was defined as the “sender” cells. The top ligands predicted to drive changes in a stromal population were defined based on the Pearson correlation coefficient between a ligand’s target prediction and observed transcriptional response, as implemented in Nichenetr. Subsequently, Nichenetr inferred the receptors and highest-predicted target genes associated with the top-ranked ligands from the ligand activity analysis.

1. Maes K, Boeckx B, Vlummens P, De Veirman K, Menu E, Vanderkerken K, et al. The genetic landscape of 5T models for multiple myeloma. Sci Rep. 2018;8(1):15030.
2. Vanderkerken K, Asosingh K, Willems A, De Raeve H, Couck P, Gorus F, et al. The 5T2MM murine model of multiple myeloma: maintenance and analysis. Methods Mol Med. 113. United States2005. p. 191-205.
3. Baryawno N, Przybylski D, Kowalczyk MS, Kfoury Y, Severe N, Gustafsson K, et al. A Cellular Taxonomy of the Bone Marrow Stroma in Homeostasis and Leukemia. Cell. 2019;177(7):1915-32.e16.
4. Kenswil KJG, Pisterzi P, Sanchez-Duffhues G, van Dijk C, Lolli A, Knuth C, et al. Endothelium-derived stromal cells contribute to hematopoietic bone marrow niche formation. Cell Stem Cell. 2021;28(4):653-70 e11.
5. Piera-Velazquez S, Jimenez SA. Endothelial to Mesenchymal Transition: Role in Physiology and in the Pathogenesis of Human Diseases. Physiol Rev. 2019;99(2):1281-324.
6. Browaeys R, Saelens W, Saeys Y. NicheNet: modeling intercellular communication by linking ligands to target genes. Nat Methods. 2020;17(2):159-62.
