# Supplementary material for: A proinflammatory response and polarized differentiation of stromal elements characterizes the murine myeloma bone marrow niche
Source: Exp Hematol Oncol. 2025 Feb 26;14:22. doi: 10.1186/s40164-025-00606-x (PMC11866767; doi:10.1186/s40164-025-00606-x)
Supplement: Supplementary file 2 — Supplementary Material 2 [file 40164_2025_606_MOESM2_ESM.pdf]

Supplementary Figure 1

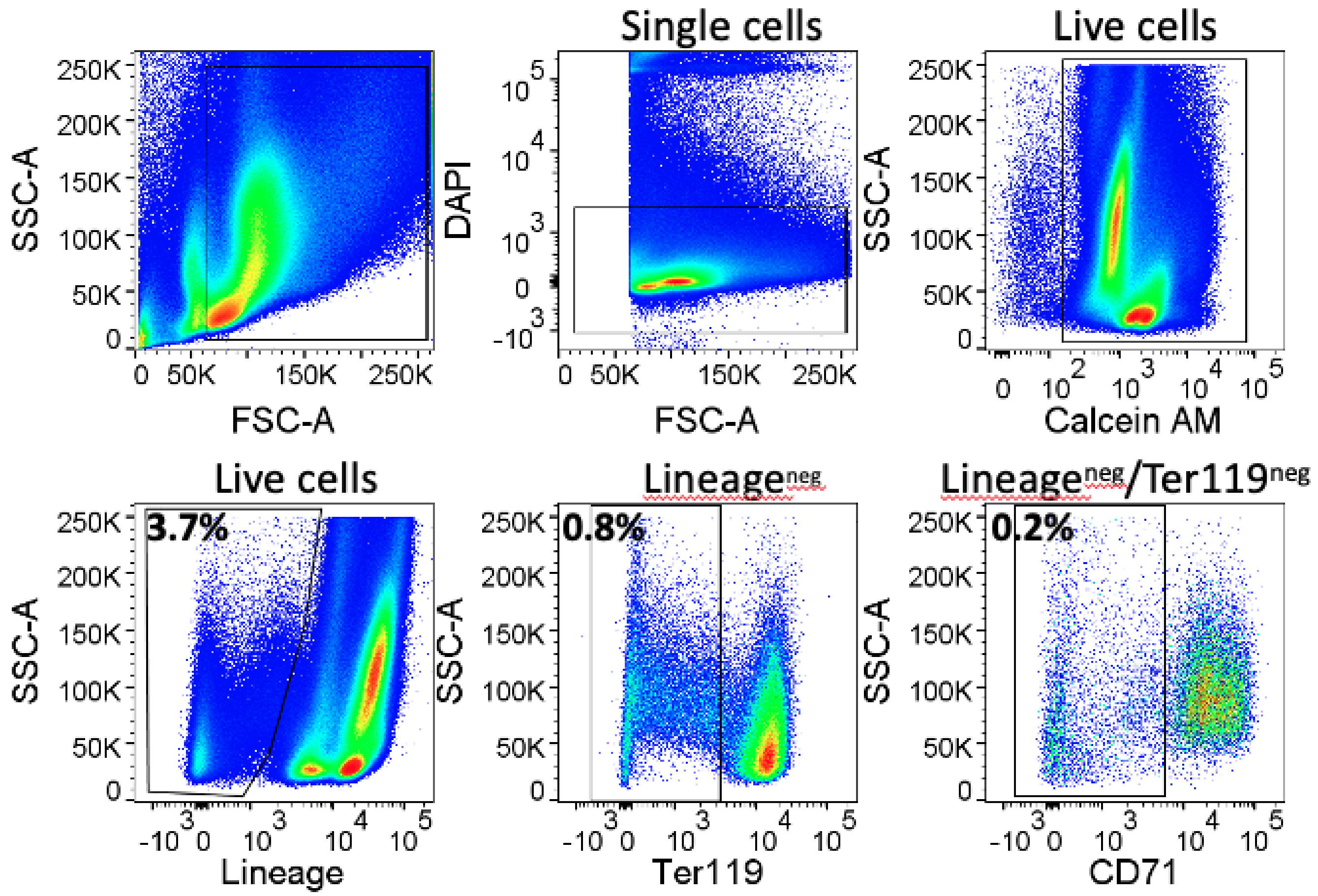

Supplementary Figure 1. Gating strategy for isolation of mouse bone marrow stromal cells (Dapi- Calcein AM+ Lin- CD71- Ter119- cells).

Supplementary Figure 2

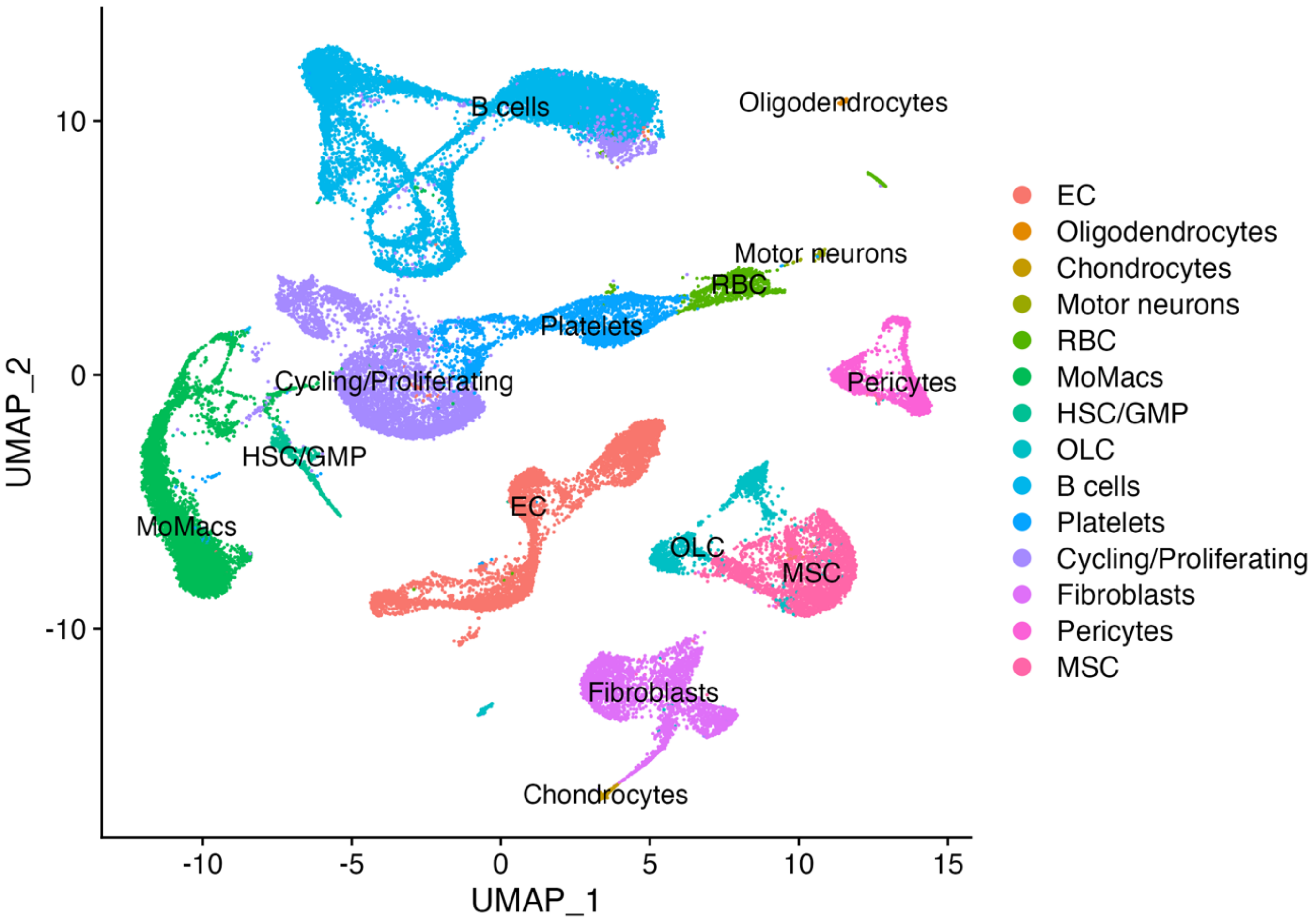

Supplementary Figure 2. UMAP of all annotated cell populations, both immune and stromal. RBCs – red blood cells; HSC/GMP - hematopoietic stem cells/granulocyte-monocyte progenitor; MoMacs – monocytes and macrophages; OLC – osteo-lineage cells; SEC – sinusoidal endothelial cells; MSC – mesenchymal stem cells; EC –endothelial cells

Supplementary Figure 3

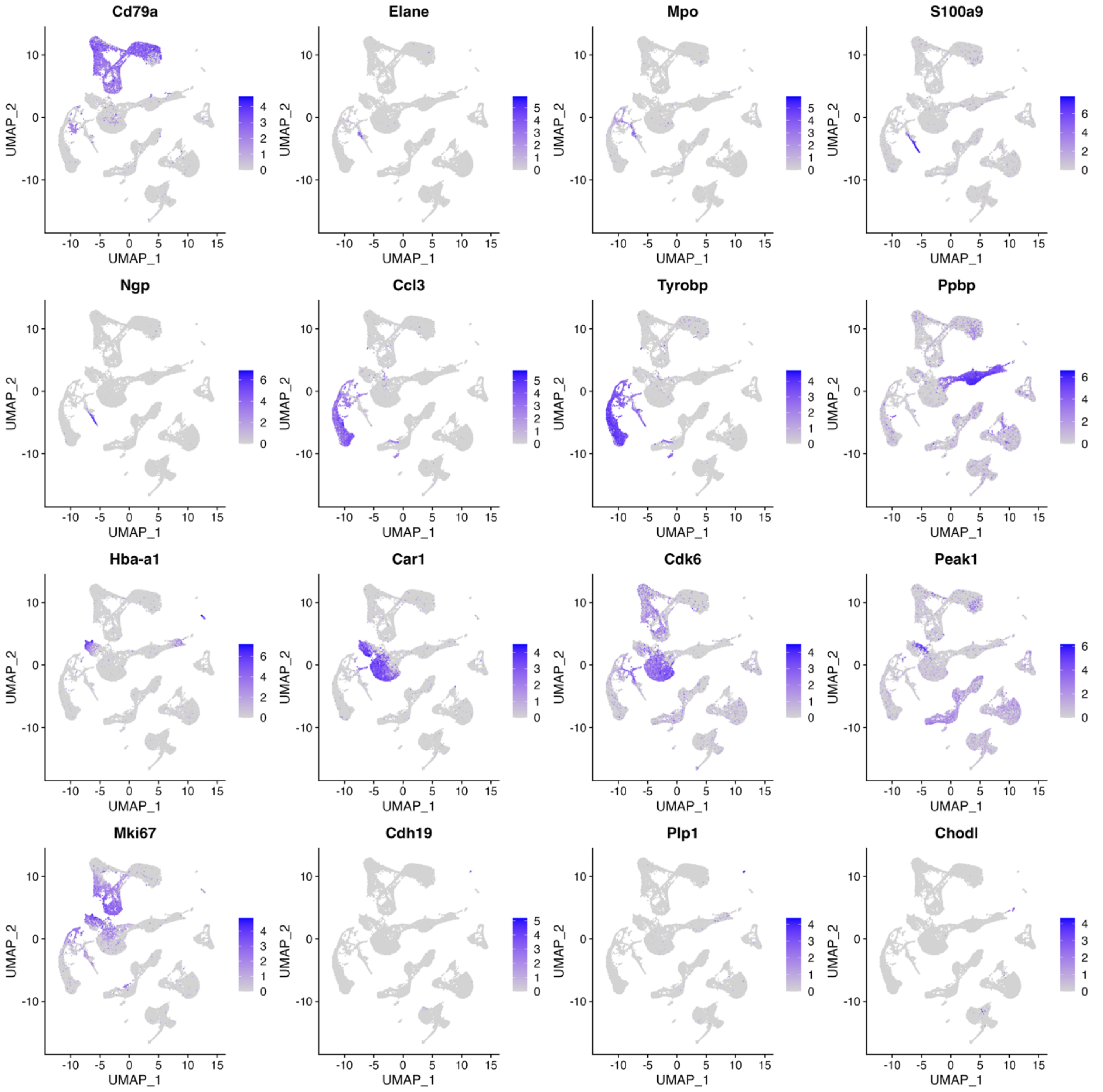

Supplementary Figure 3. Feature plots showing expression of marker genes used for annotating the non-stromal cell populations. Cell type-marker gene relationships as follows: B-cells - *Cd79a*; hematopoietic stem cells/granulocyte-monocyte progenitor (HSC/GMP) – *Elane*, *Mpo*, *S100a9*, *Ngp*; monocyte/macrophages (MoMacs) – *Ccl3*, *Tyrobp*; Platelets – *Ppbp*; red blood cells (RBC) – *Hba-a1*; cycling/proliferating cells – *Car1*, *Cdk6*, *Peak1*, *Mki67*; oligodendrocytes – *Cdh19*, *Plp1*; motor neurons - *Chodl*

Supplementary Figure 4

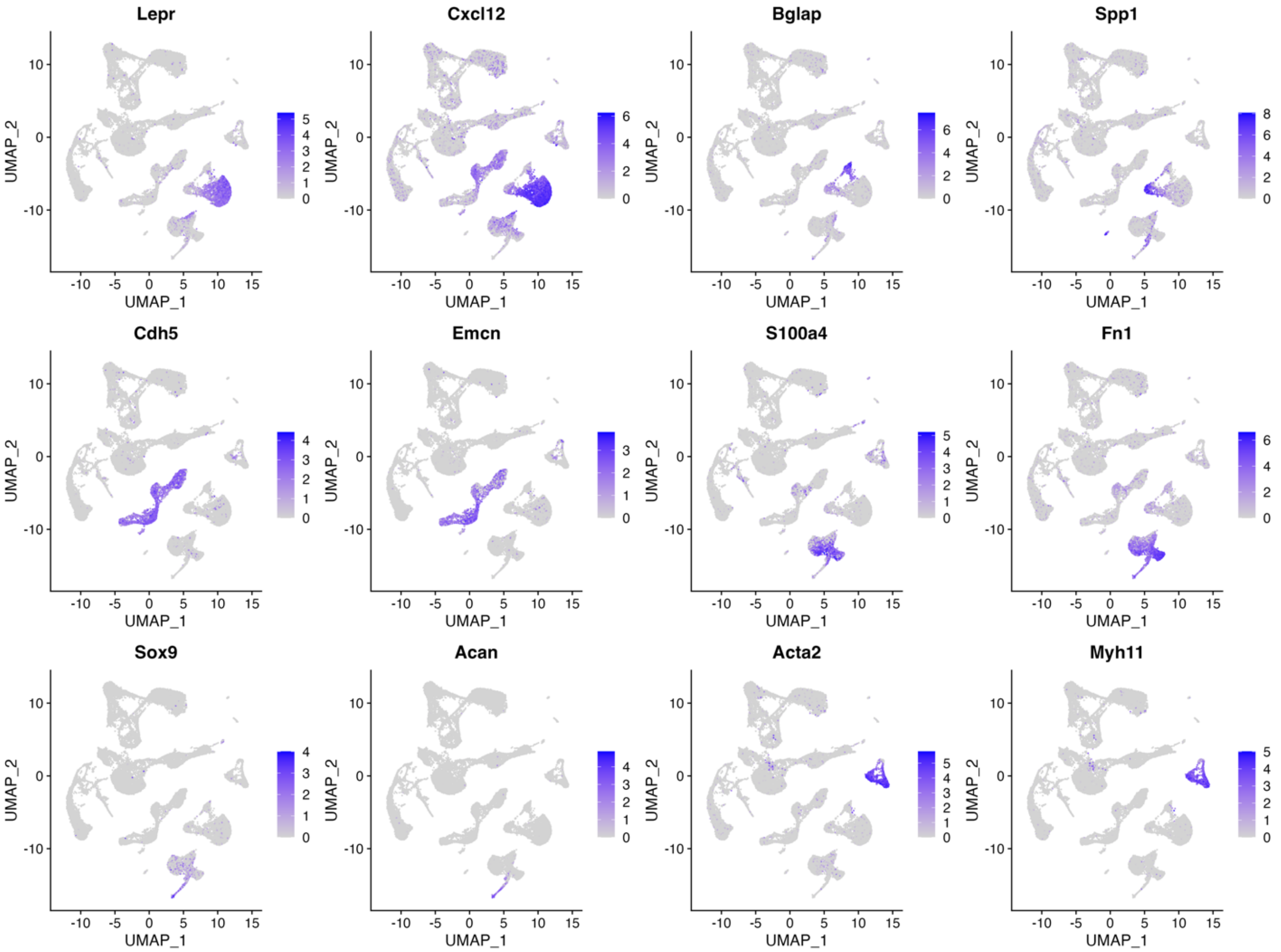

Supplementary Figure 4. Feature plots showing expression of marker genes used for annotating the stromal cell populations. Cell type-marker gene relationships as follows: mesenchymal stem cells (MSC) – *Lepr*, *Cxcl12*; osteo-lineage cells (OLC) – *Bglap*, *Spp1*; endothelial cells (EC) – *Cdh5*, *Emcn*; fibroblasts – *S100a4*, *Fn1*; chondrocytes – *Sox9*, *Acan*; pericytes – *Acta2*, *Myh11*

Supplementary Figure 5

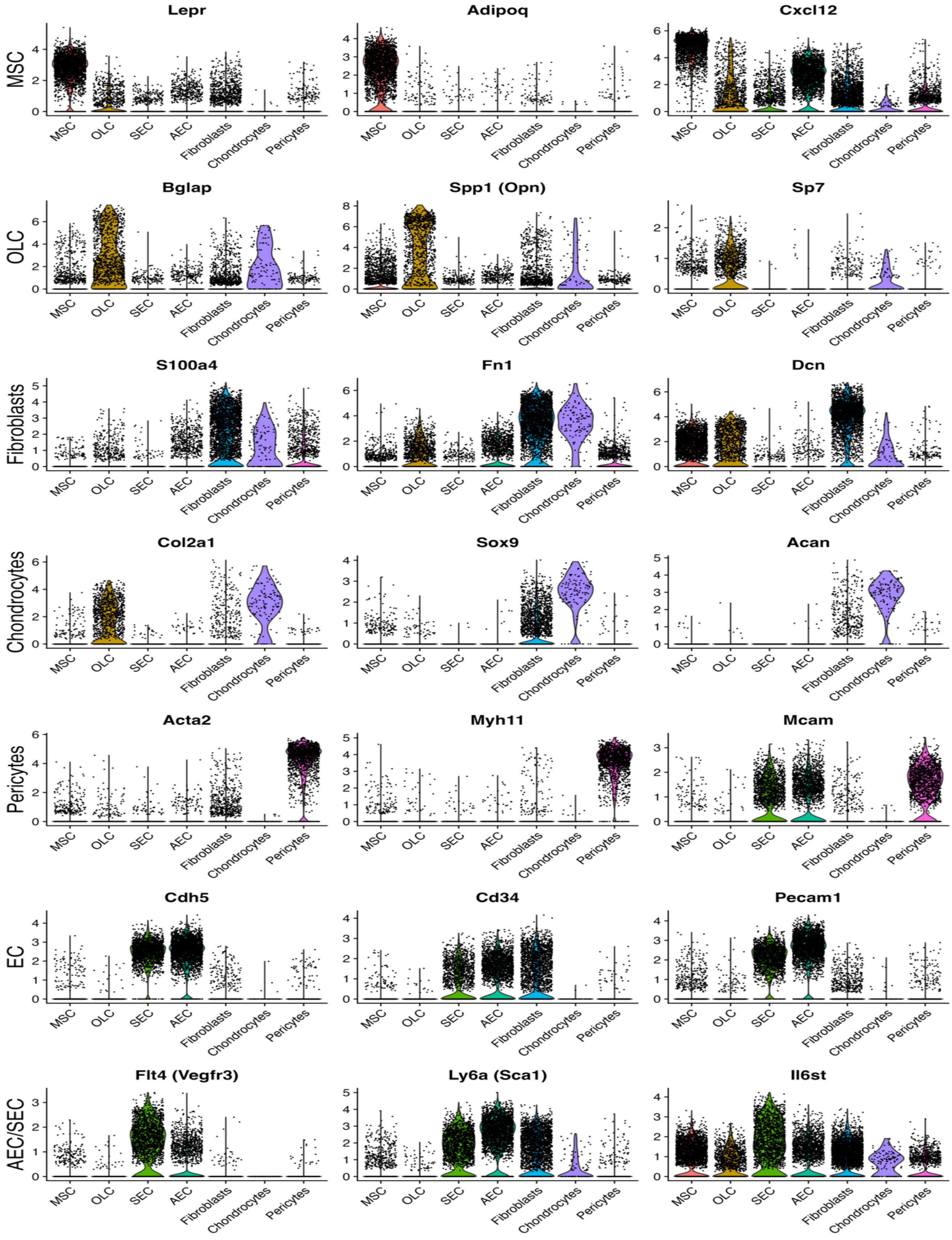

Supplementary Figure 5. Violin plots showing expression of the marker genes used to annotate the stromal populations. MSC – mesenchymal stem cells; OLC – osteo-lineage cells; EC – endothelial cells; AEC – arterial endothelial cells; SEC – sinusoidal endothelial cells.

Supplementary Figure 6

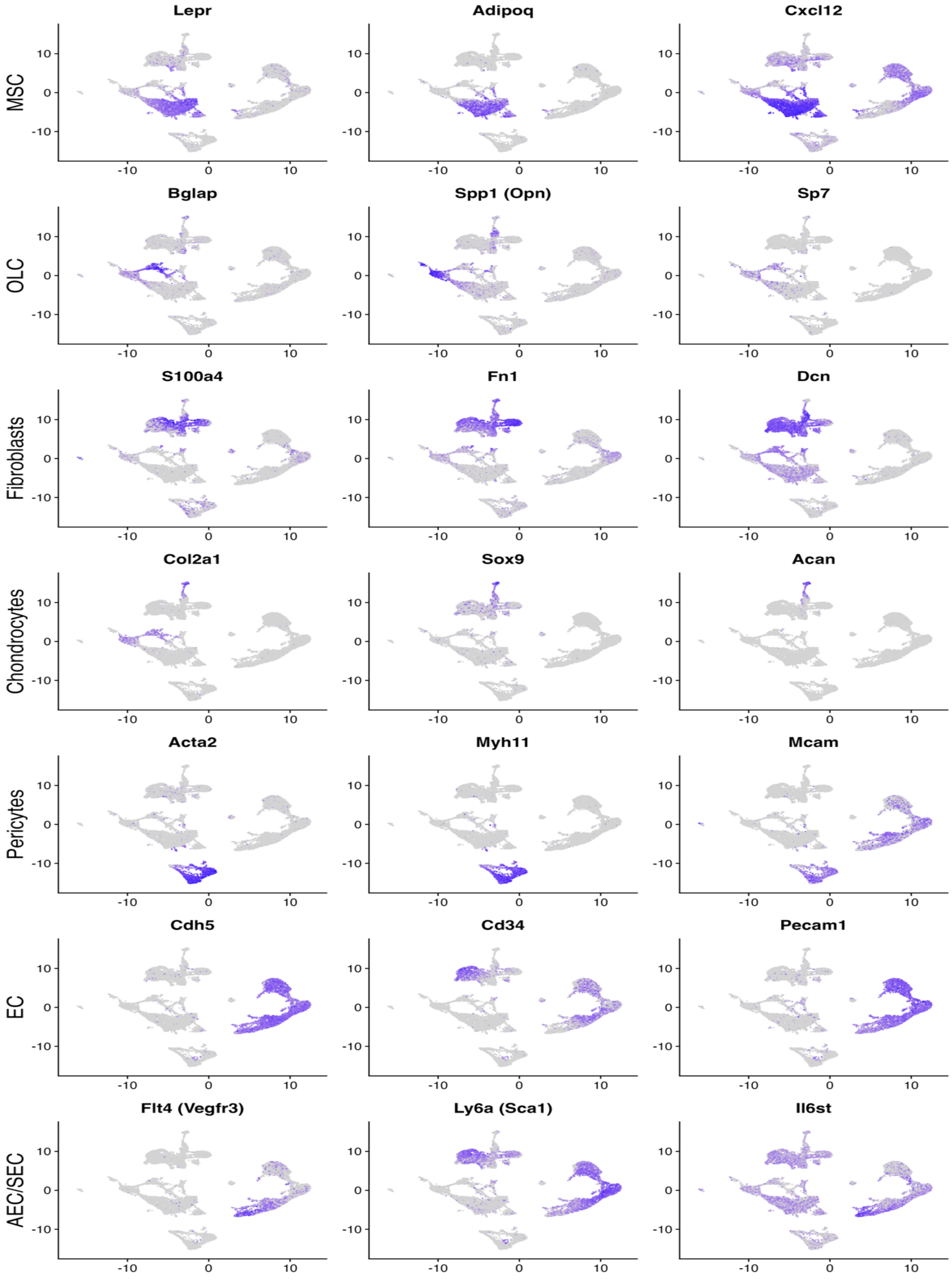

Supplementary Figure 6. Feature plots showing expression of the marker genes used to annotate the stromal populations. MSC – mesenchymal stem cells; OLC – osteo-lineage cells; EC – endothelial cells; AEC – arterial endothelial cells; SEC – sinusoidal endothelial cells.

Supplementary Figure 7

A.

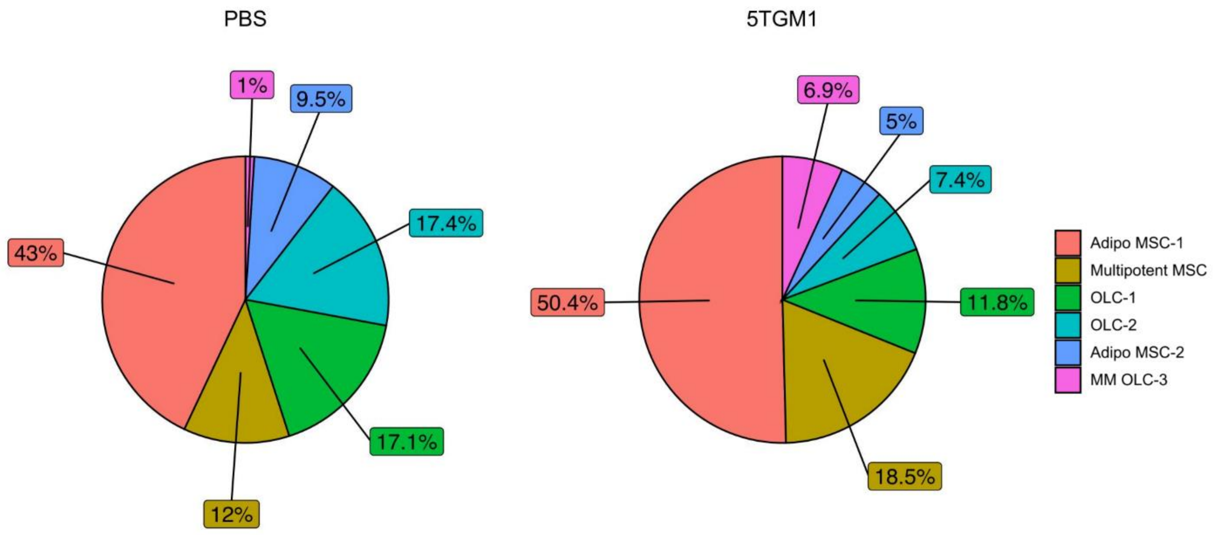

B.

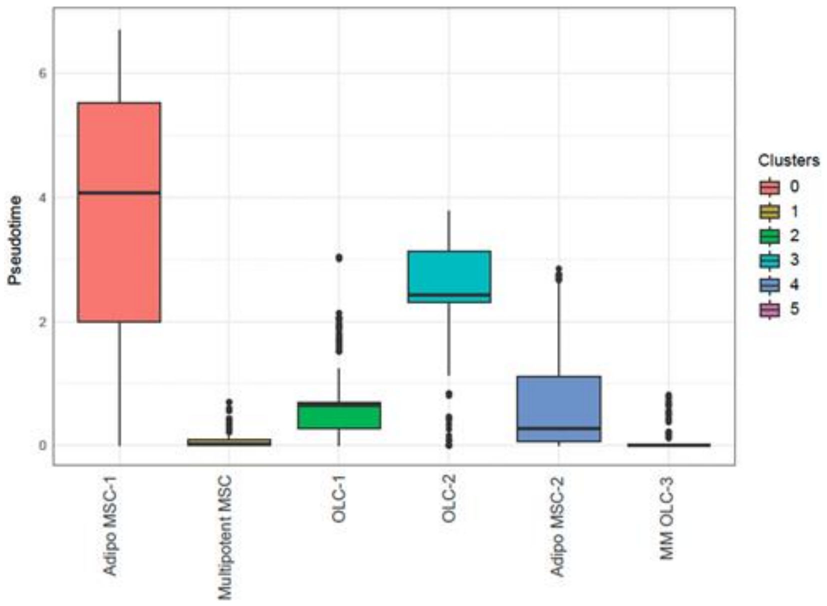

Supplementary Figure 7. A. Relative abundances of MSC-lineage subclusters split by experimental condition. B. Box plot showing pseudotime values of each subcluster.

Supplementary Figure 8

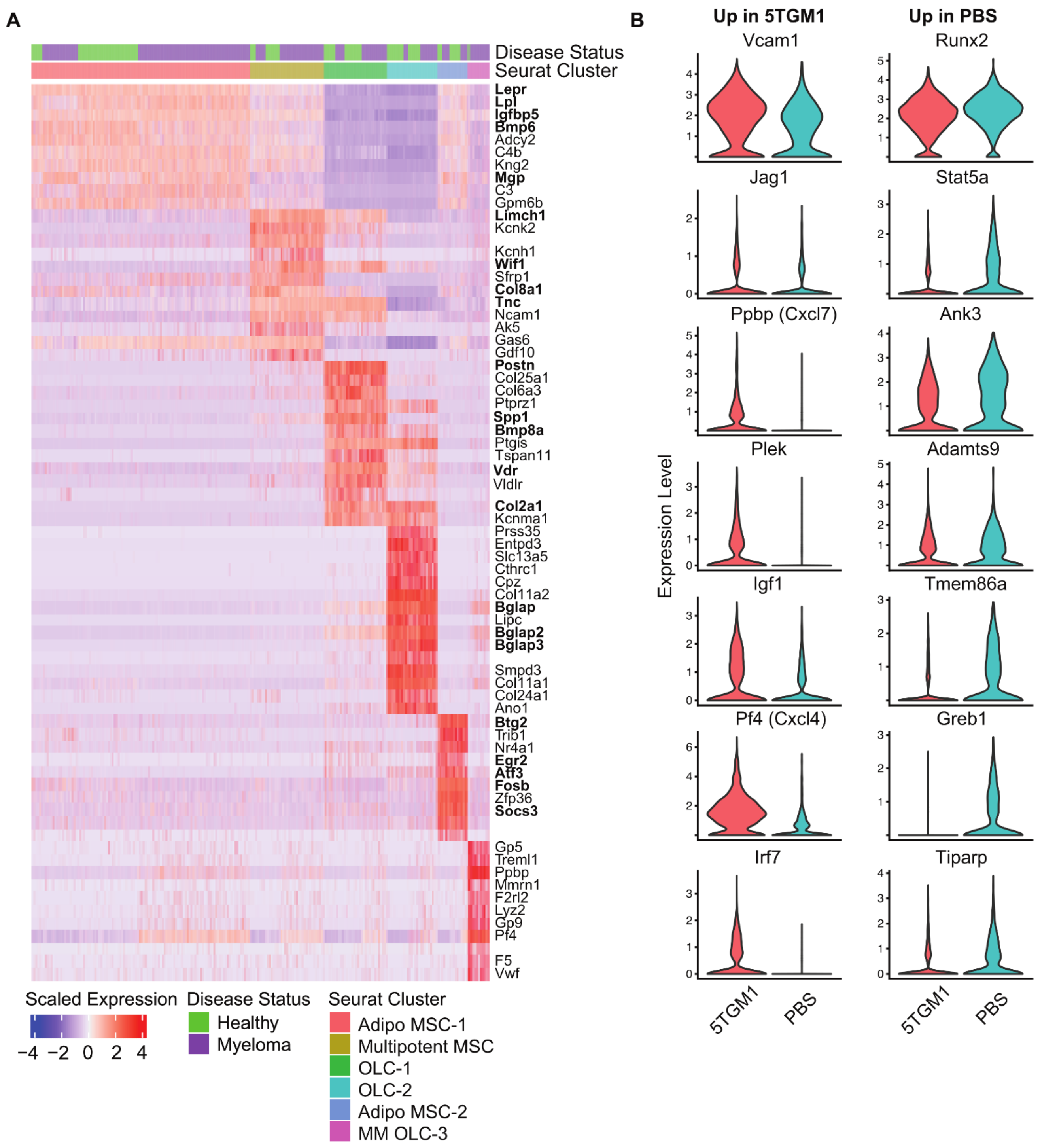

Supplementary Figure 8. A. Heatmap of the top genes differentially expressed between the 6 MSC-lineage subclusters, sorted by adjusted p-value. B. Violin plots showing 8 of the most differentially expressed between the PBS control and 5TGM1 conditions in the MSC-lineage.

Supplementary Figure 9

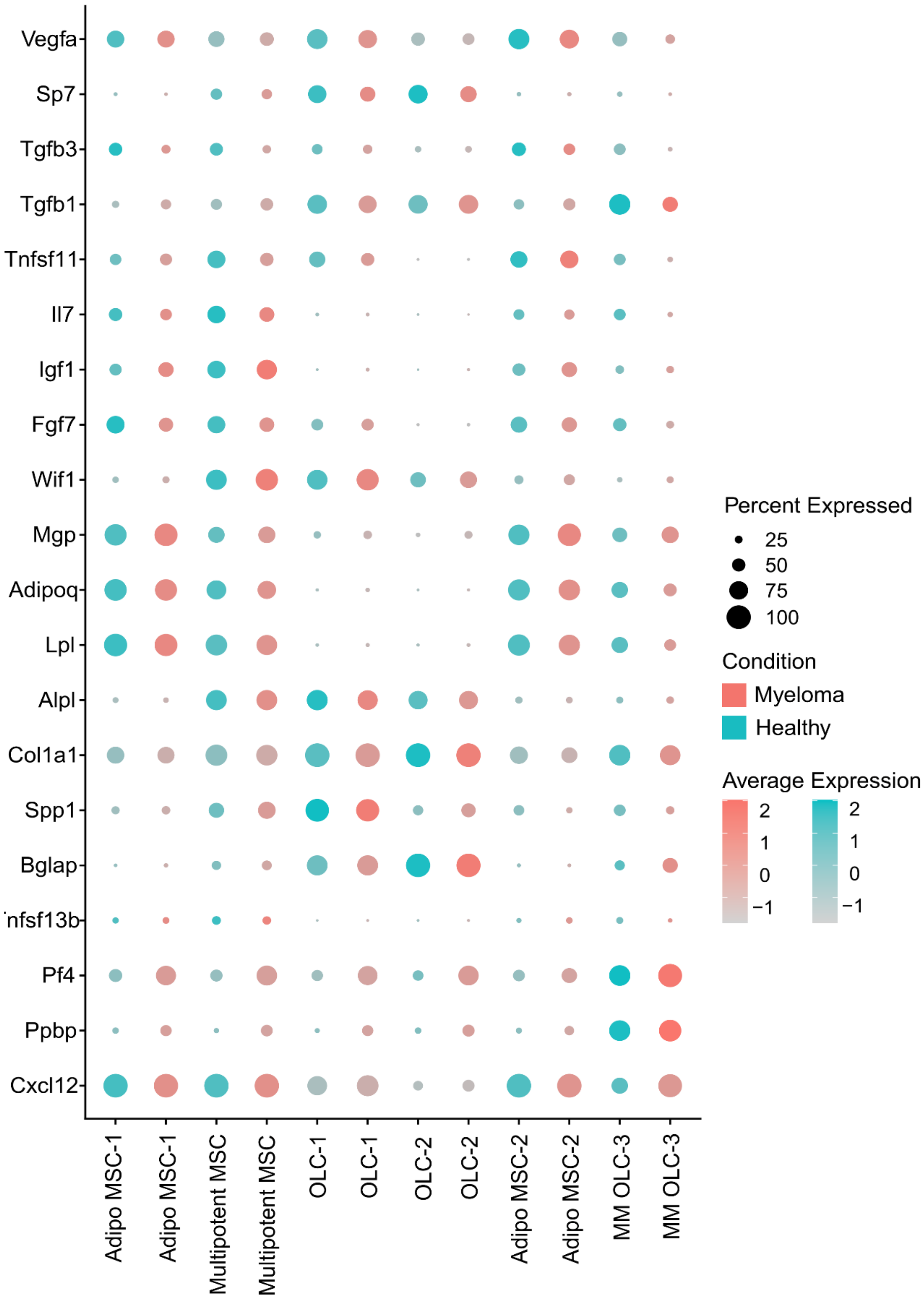

Supplementary Figure 9. Dot plot showing expression of relevant secreted molecules expressed in MSC-lineage subcluster, split by condition. C0 refers to cluster 0 (Adipo MSC-1), C1 refers to cluster 1 (Multipotent MSC), C2 refers to cluster 2 (OLC-1), C3 refers to cluster 3 (OLC-2), C4 refers to cluster 4 (Adipo MSC-2), and C5 refers to cluster 5 (MM OLC-3).

Supplementary Figure 10

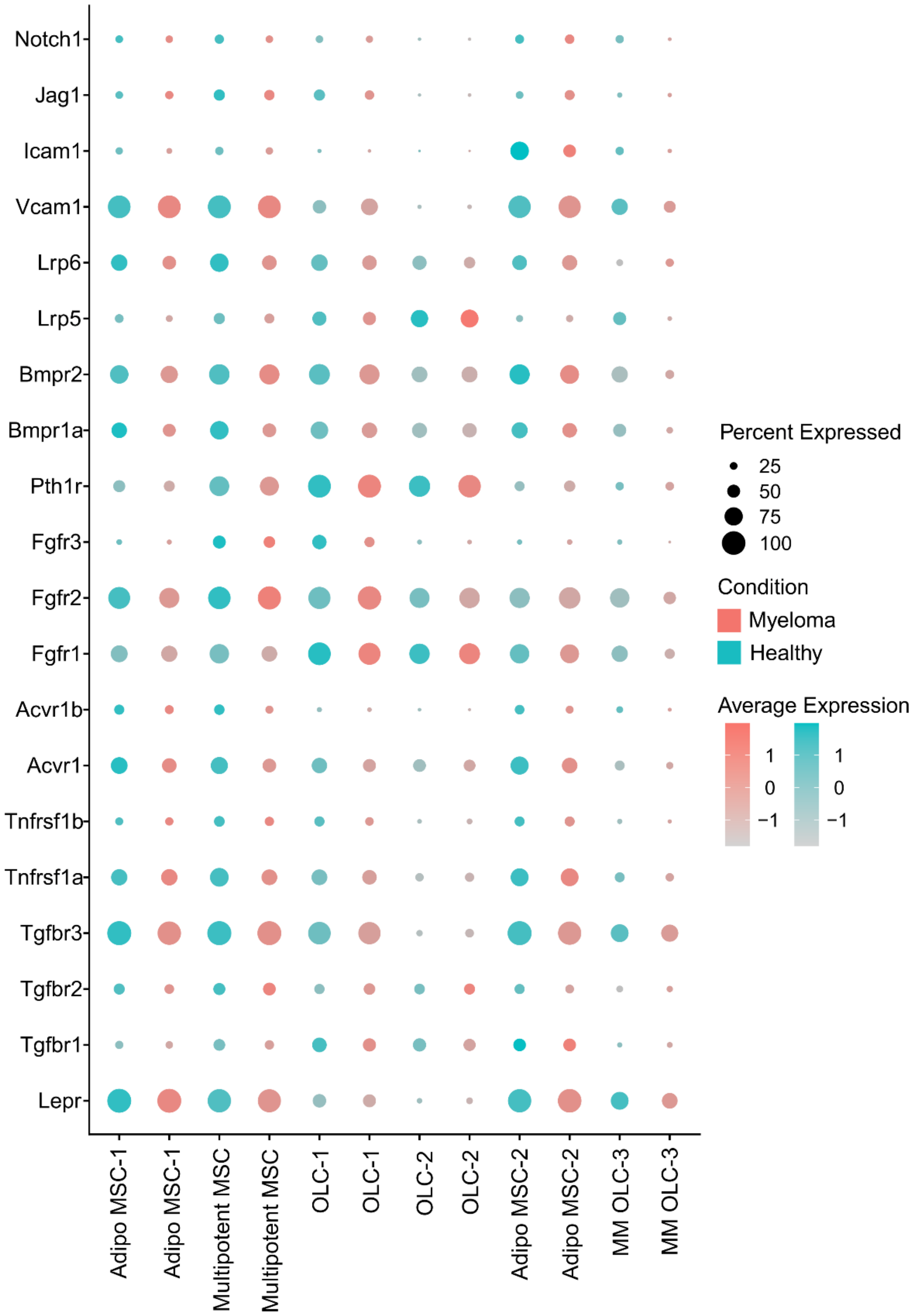

Supplementary Figure 10. Dot plot showing expression of relevant receptor and adhesion molecules expressed in MSC-lineage subclusters, split by condition. C0 refers to cluster 0 (Adipo MSC-1), C1 refers to cluster 1 (Multipotent MSC), C2 refers to cluster 2 (OLC-1), C3 refers to cluster 3 (OLC-2), C4 refers to cluster 4 (Adipo MSC-2), and C5 refers to cluster 5 (MM OLC-3).

Supplementary Figure 11

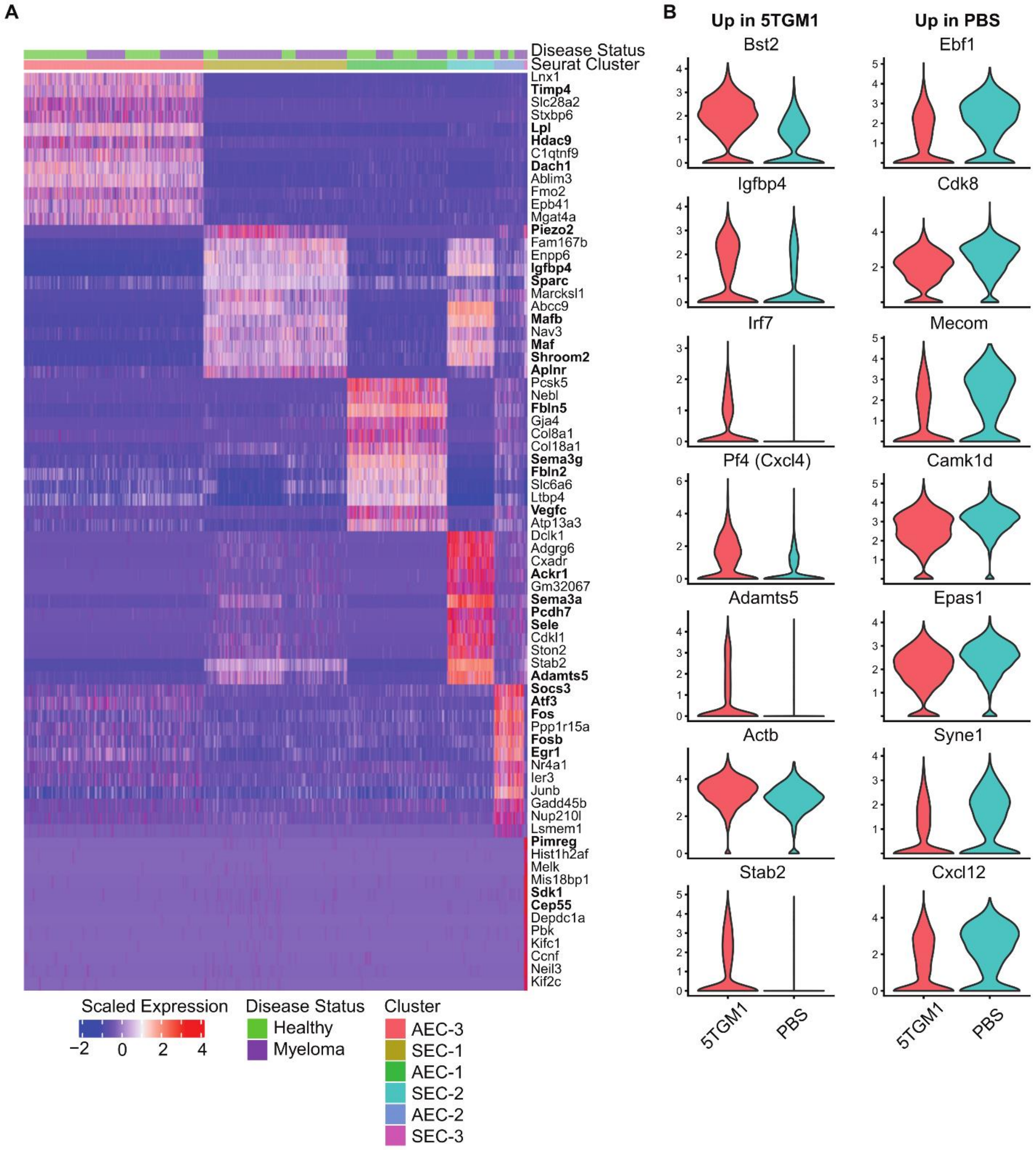

Supplementary Figure 11. A. Heatmap of the top genes differentially expressed between the 6 BMEC subclusters. B. Violin plots showing 8 of the most differentially expressed between the PBS control and 5TGM1 conditions in BMECs.

Supplementary Figure 12

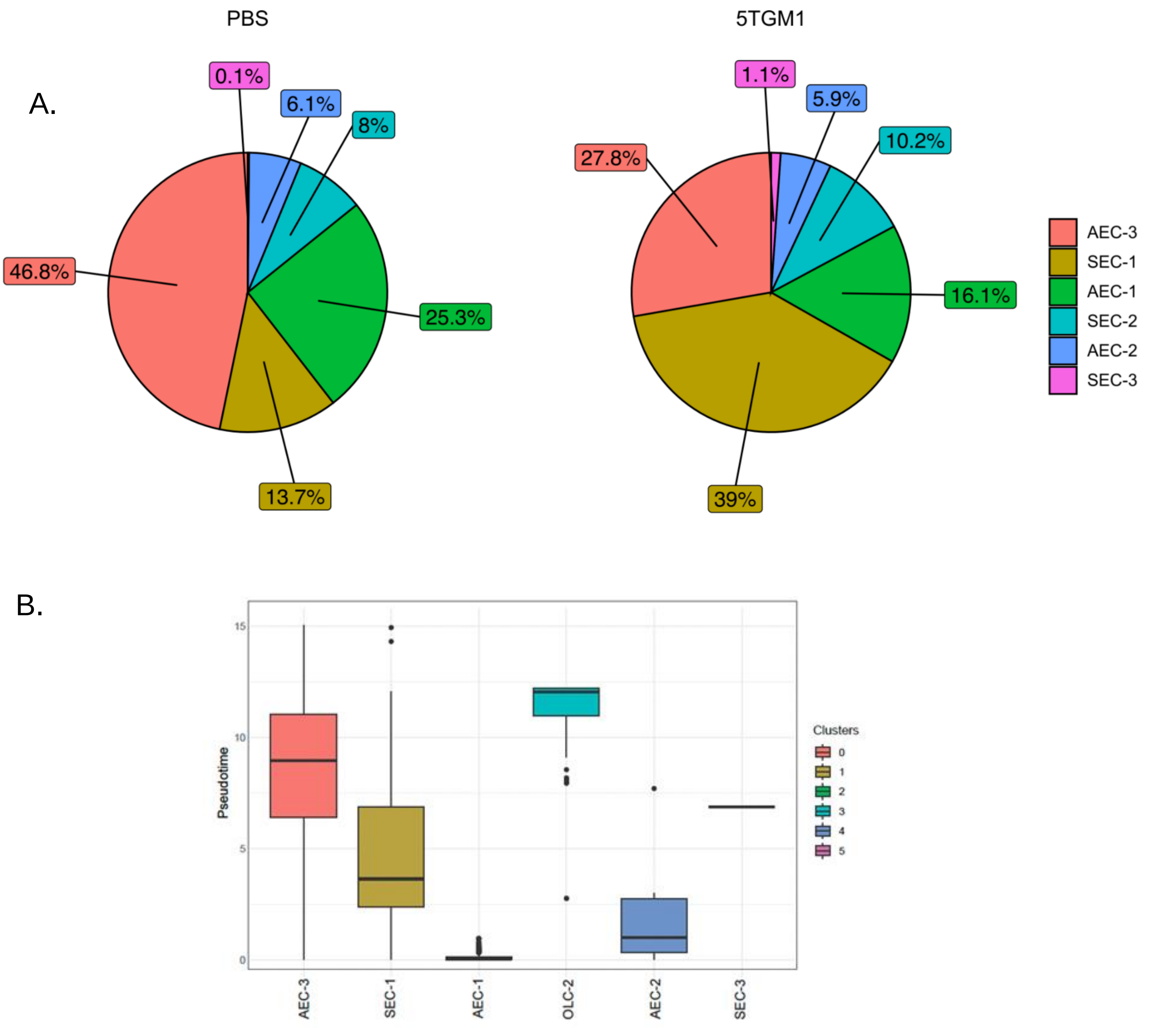

Supplementary Figure 12. A. Pie charts showing relative abundances of BMECs subclusters split by experimental condition. B. Box plot showing pseudotime values of each subcluster.

Supplementary Figure 13

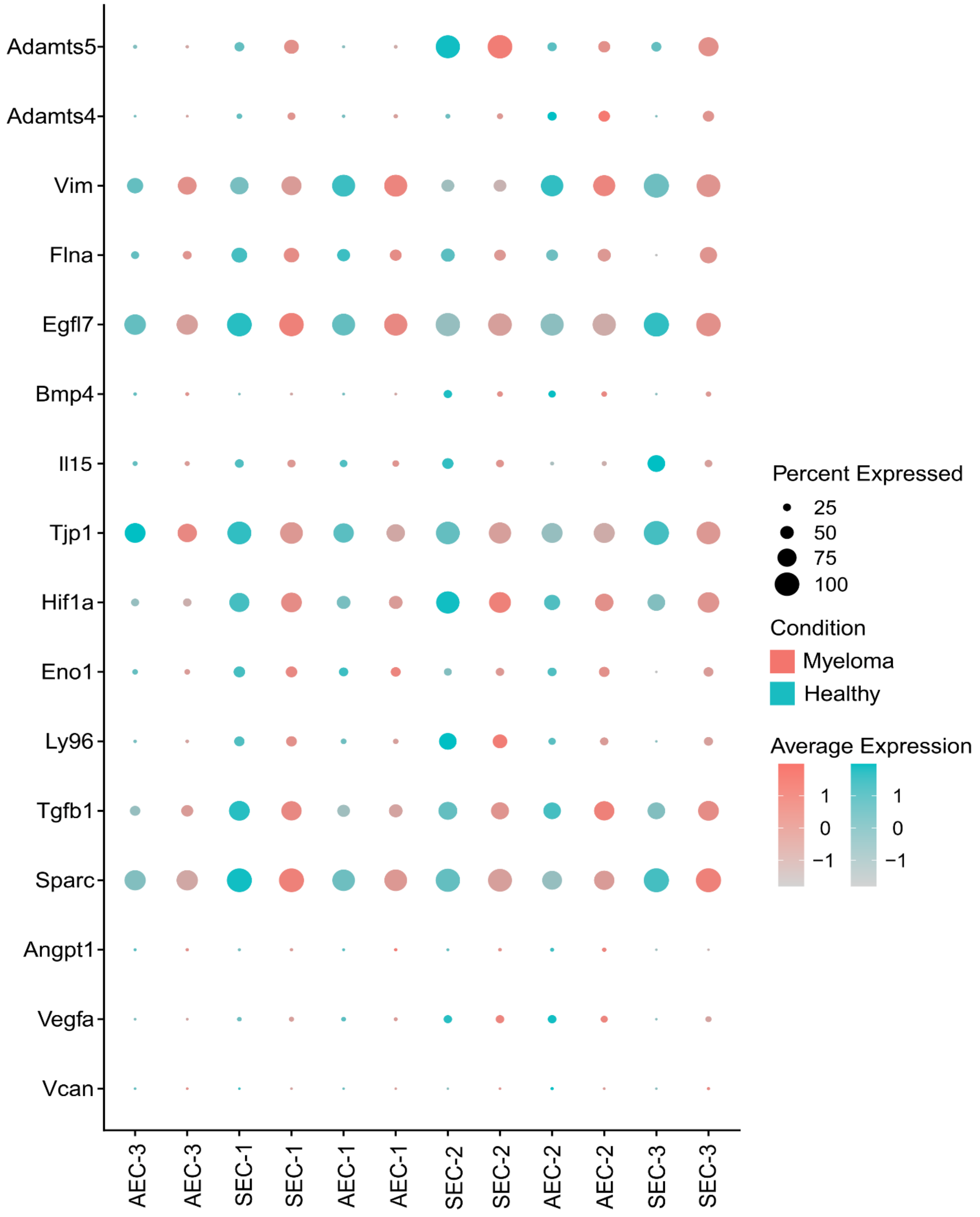

Supplementary Figure 13. Dot plot showing expression of relevant secreted molecules across BMEC subclusters, split by condition. C0 refers to cluster 0 (AEC-3), C1 refers to cluster 1 (SEC-1), C2 refers to cluster 2 (AEC-1), C3 refers to cluster 3 (SEC-2), C4 refers to cluster 4 (AEC-2), and C5 refers to cluster 5 (SEC-3).

Supplementary Figure 14

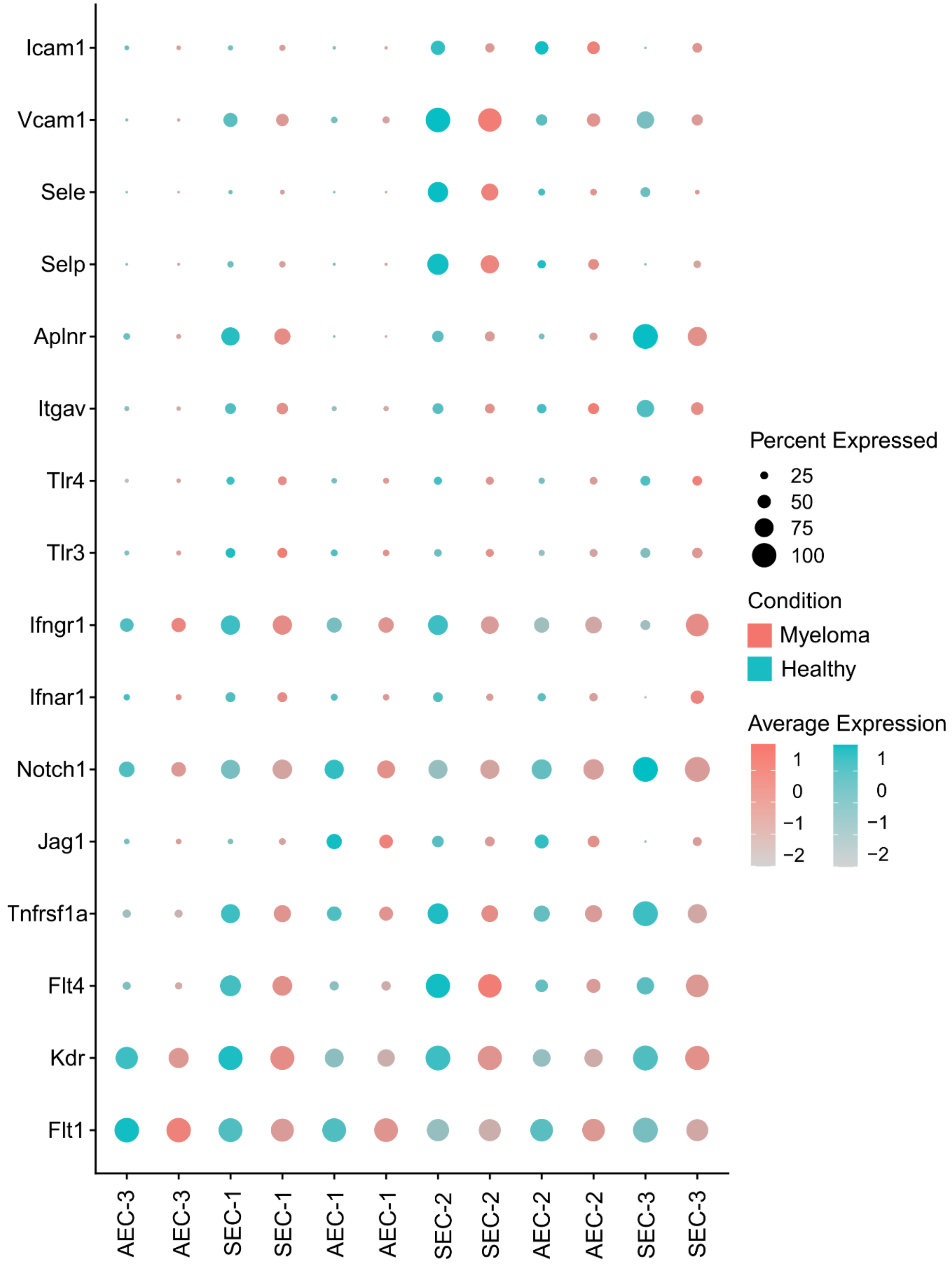

Supplementary Figure 14. Dot plot showing expression of relevant receptor and adhesion molecules across BMEC subclusters, split by condition. C0 refers to cluster 0 (AEC-3), C1 refers to cluster 1 (SEC-1), C2 refers to cluster 2 (AEC-1), C3 refers to cluster 3 (SEC-2), C4 refers to cluster 4 (AEC-2), and C5 refers to cluster 5 (SEC-3).

Supplementary Figure 15

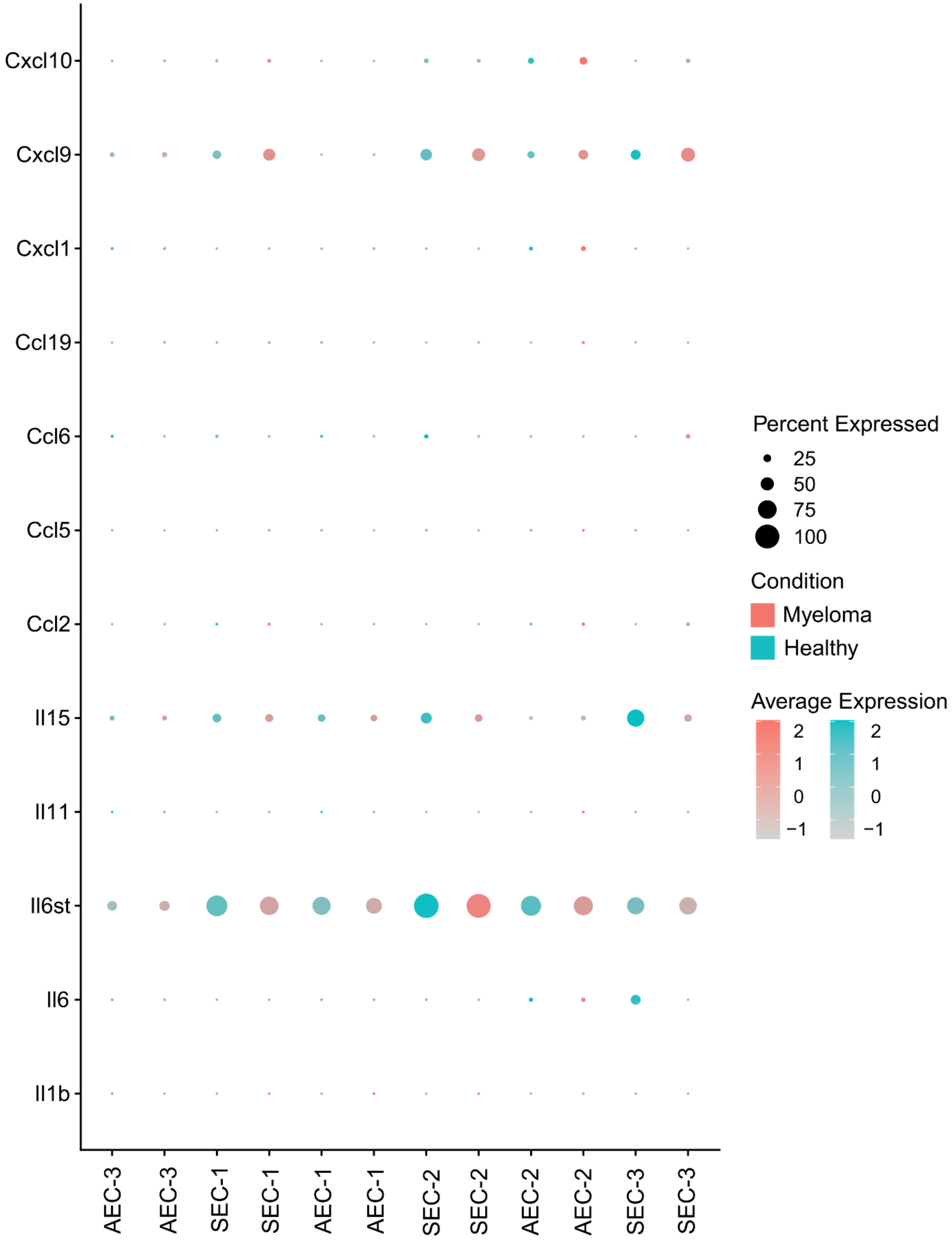

Supplementary Figure 15. Dot plots showing expression of selected proinflammatory cytokines across BMEC subclusters, split by condition. C0 refers to cluster 0 (AEC-3), C1 refers to cluster 1 (SEC-1), C2 refers to cluster 2 (AEC-1), C3 refers to cluster 3 (SEC-2), C4 refers to cluster 4 (AEC-2), and C5 refers to cluster 5 (SEC-3).

Supplementary Figure 16

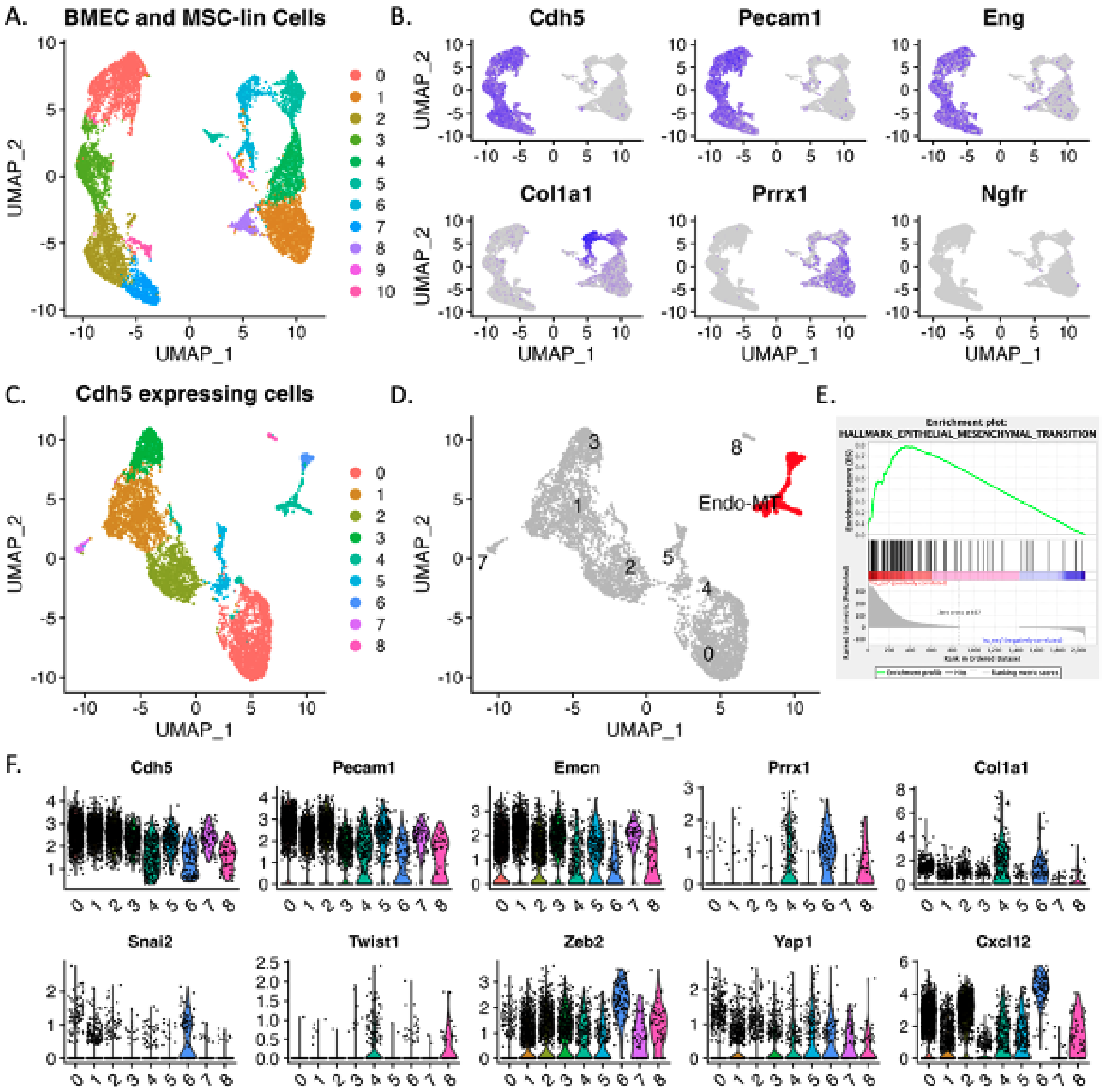

Supplementary Figure 16. Analysis of cells undergoing endothelial-mesenchymal transformation (EndoMT). A. UMAP of subclustered BMEC and MSC-lineage cells. B. Feature plots showing expression of endothelial and mesenchymal cell markers. C. UMAP of BMEC and MSC-lineage cells expressing Cdh5. D. UMAP showing Cdh5 positive cells and highlighting putative EndoMT cells. E. Violin plots showing expression of key EndoMT genes across Cdh5 positive cell clusters.

Supplementary Figure 17

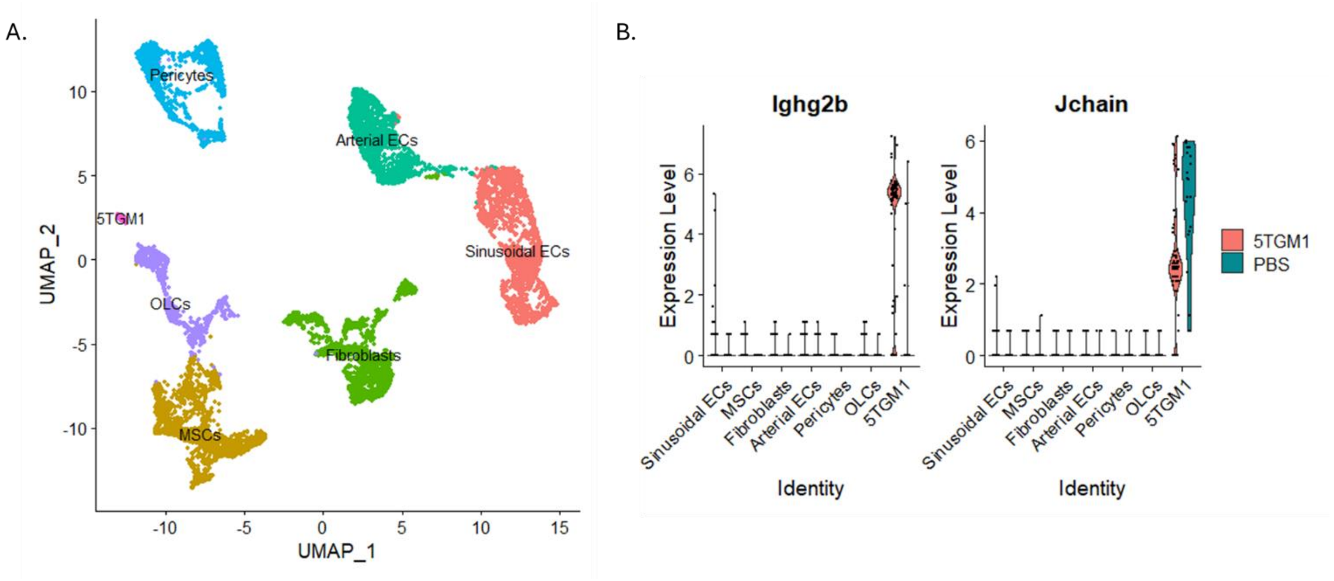

Supplementary Figure 17. A. UMAP of the primary stromal populations and 5TGM1 cells. B. Violin plots showing expression of Ighg2b and Jchain genes in the 5TGM1 cluster.

Supplementary Figure 18

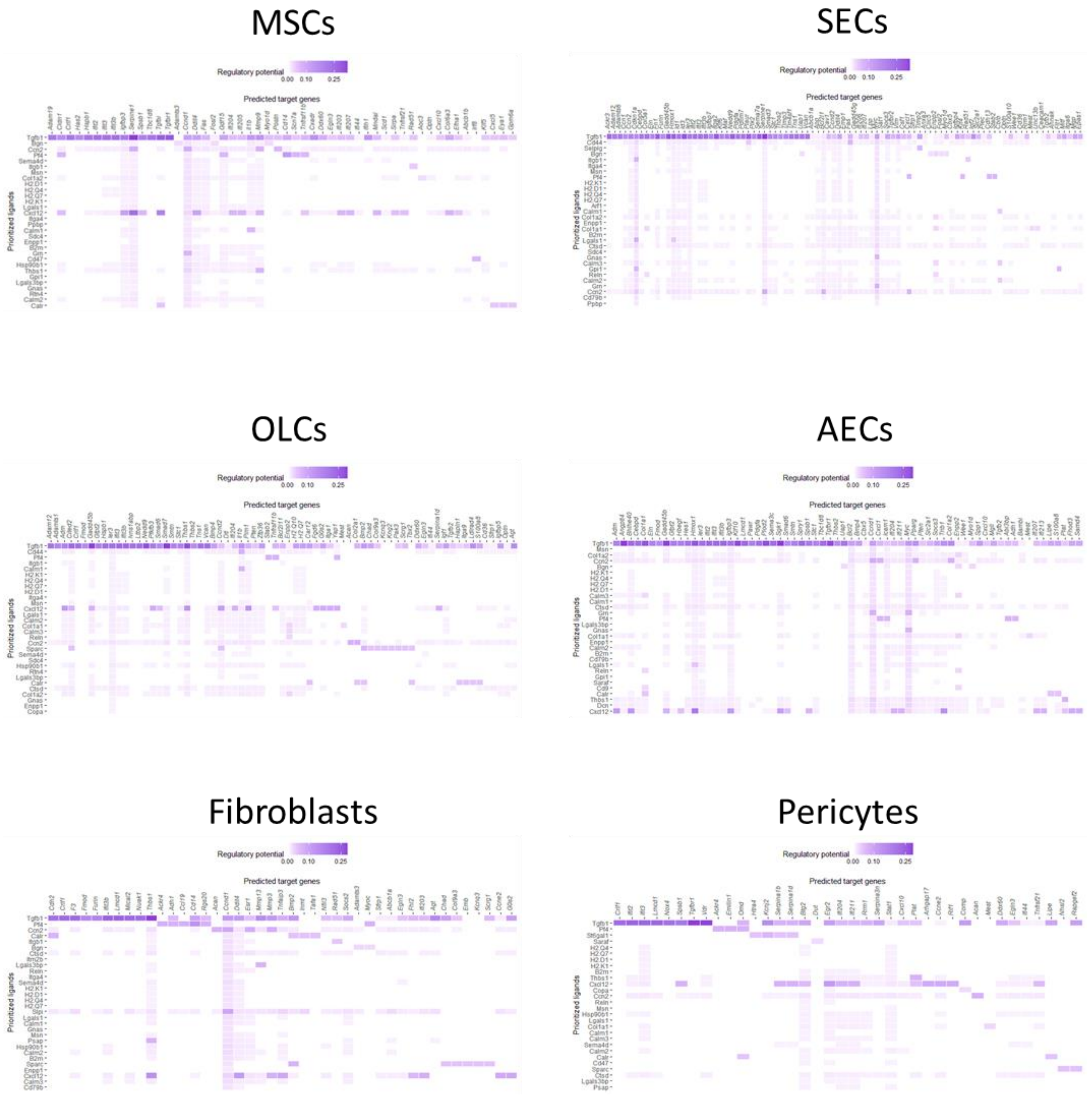

Supplementary Figure 18. Top-predicted target genes of ligands that are top-ranked in the ligand activity analysis. The Y-axis represents the prioritized ligands within sender cells (5TGM1), while the X-axis illustrates the active target genes within the receiver cells (MSCs, Fibroblasts, Arterial ECs (AECs), Pericytes, Sinusoidal ECs (SECs) and OLCs).

Supplementary Figure 19

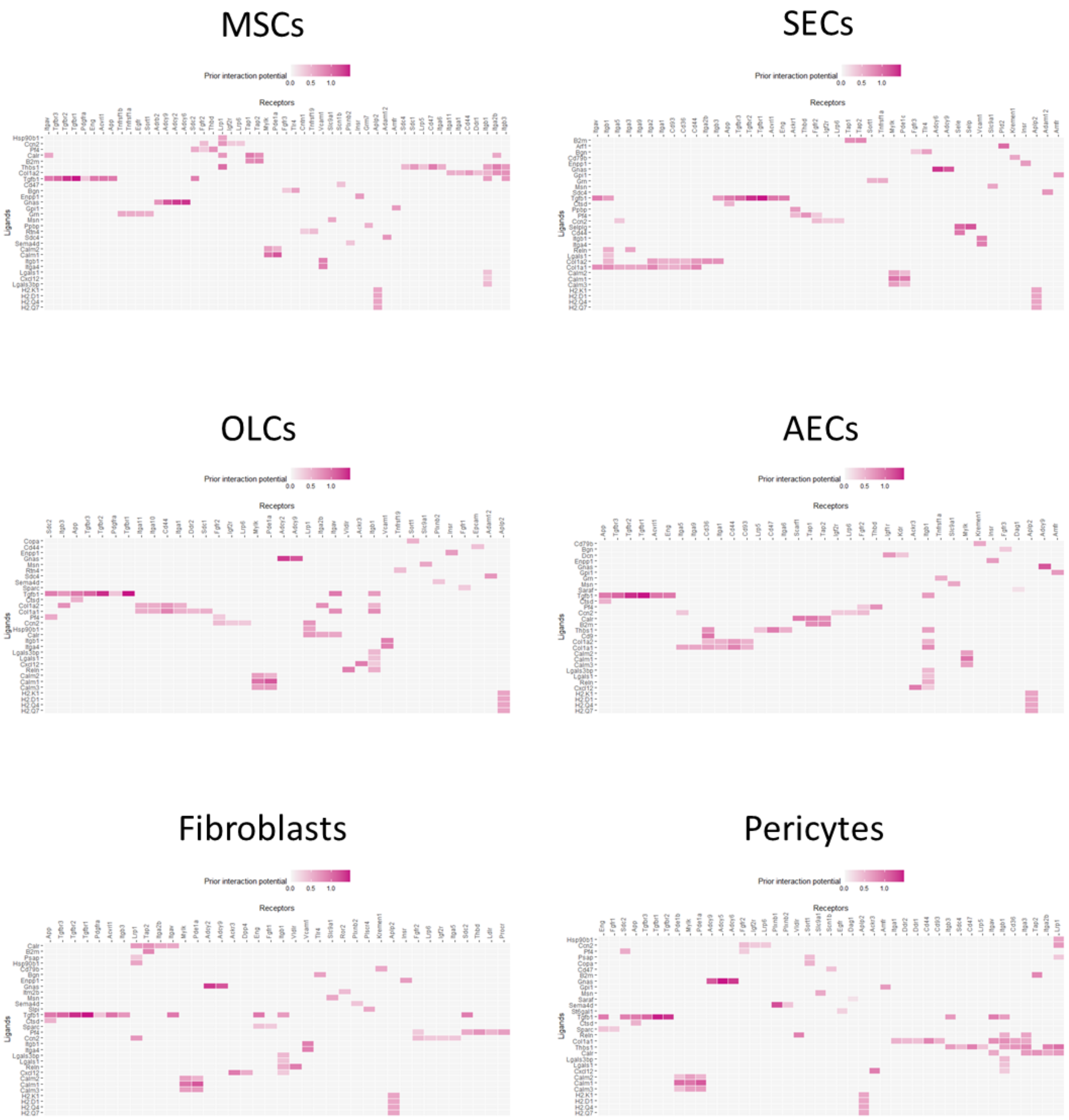

Supplementary Figure 19. Receptors of the top-ranked ligands in the ligand activity analysis. The Y-axis represents the ligands in the sender cells (5TGM1) while the X-axis illustrates the receptors in the receiver cells (MSCs, Fibroblasts, Arterial ECs, Pericytes, Sinusoidal ECs and OLCs).

Supplementary Figure 20

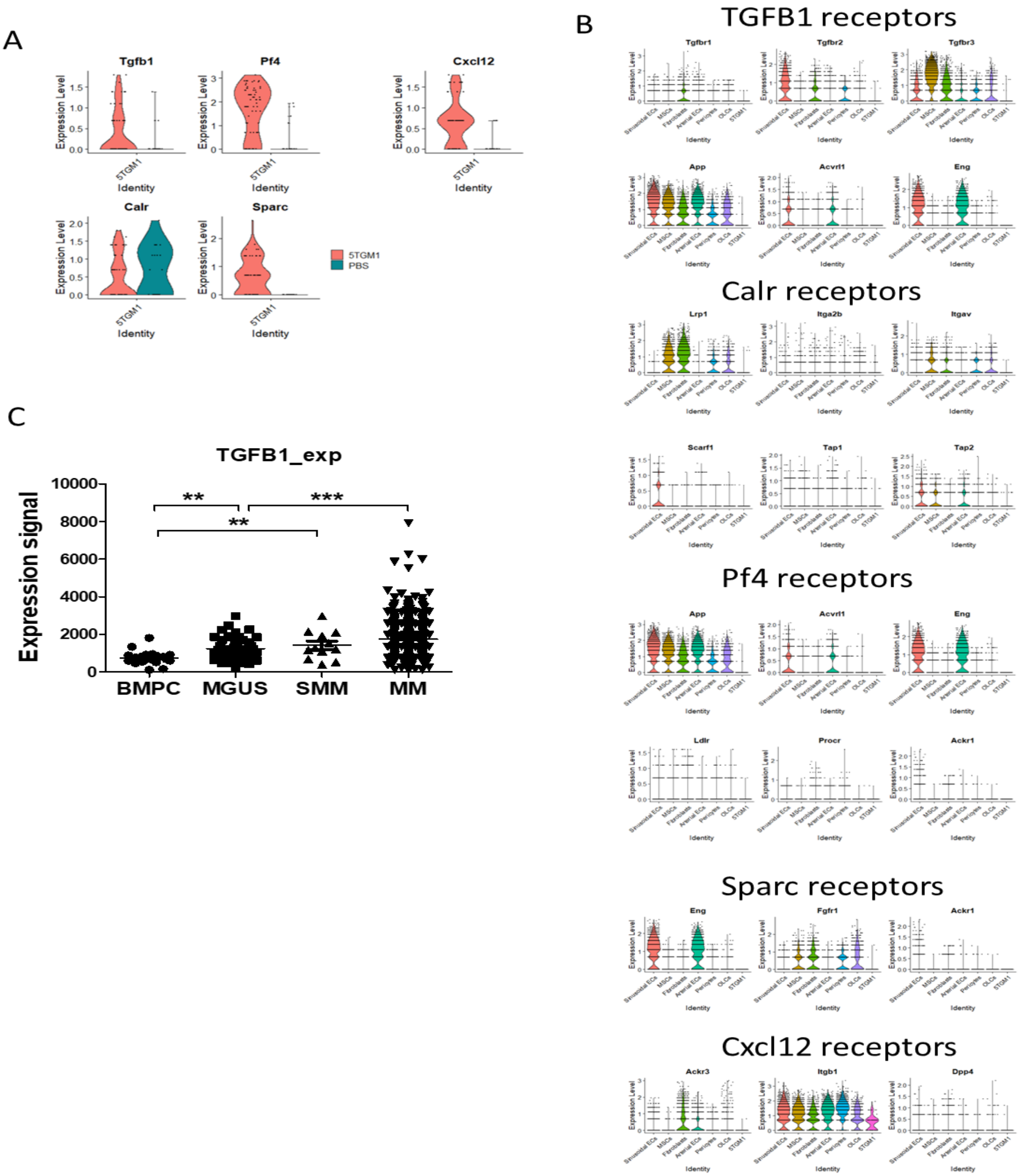

Supplementary Figure 20. The expression of identified ligands and receptors. A. Tgfb1, Pf4 (Cxcl4), Cxcl12, Calr and Sparc expression in 5TGM1 cells. B. The receptors of Tgfb1, Pf4 (Cxcl4), Cxcl12, Calr and Sparc expression in stromal cells. C. TGFB1 expression in human MM cells. BMPC: Bone Marrow Plasma Cells, MGUS: Monoclonal Gammopathy of Undetermined Significance, SMM: Smoldering Multiple Myeloma, MM: Multiple Myeloma.
