# Supplementary material for: A proinflammatory response and polarized differentiation of stromal elements characterizes the murine myeloma bone marrow niche
Source: Exp Hematol Oncol. 2025 Feb 26;14:22. doi: 10.1186/s40164-025-00606-x (PMC11866767; doi:10.1186/s40164-025-00606-x)
Supplement: Supplementary file 3 — Supplementary Material 3 [file 40164_2025_606_MOESM3_ESM.pdf]

Supplementary Table1. Absolute cell numbers of the bone marrow (BM) and sorted Alive lineage- Ter119- CD71- cells and CellRanger outputs.

Mouse ID	Analysis - week number after injection	Analysis - days after injection	Total BM cell number x 10^6	Sorted Stromal cell number: Alive Lineage- Ter119- CD71-	CellRanger cell number outputs
PBS1	Week 7	48 days	36	33,000	28,407
PBS2			30	25,000	
PBS3			32	26,000	
PBS4	Week 8	55 days	42	25,000	
5TGM1-815R	Week 7	48 days	39	36,000	25,051
5TGM1-813RR			35	25,000	
5TGM1-815B			40	23,000	
5TGM1-815RR	Week 8	55 days	40	30,000	

Supplementary Table 2
Top GSEA Genes in MM Stromal Cells

Pathway	Gene	Gene Rank	Running ES
INTERFERON ALPHA RESPONSE	Ifitm3	5	0.1422
	Irf7	8	0.2573
	Isg15	12	0.3504
	Rtp4	13	0.4415
	Bst2	22	0.5099
	B2m	30	0.5608
	Lgals3bp	38	0.6062
	Ifi27	44	0.6466
	Parp14	93	0.6641
	Herc6	95	0.6921
INTERFERSON GAMMA RESPONSE	Ifitm3	5	0.1138
	Irf7	8	0.2060
	Isg15	12	0.2805
	Rtp4	13	0.3536
	Xaf1	20	0.4126
	Bst2	22	0.4687
	B2m	30	0.5091
	Lgals3bp	38	0.5453
	Ifi27	44	0.5774
	Stat1	54	0.6072
OXIDATIVE PHOSPHORYLATION	Gpx4	32	0.0786
	Ldha	85	0.1145
	Cox5a	119	0.1478
	Etfb	125	0.1843
	Cyb5a	142	0.2160
	Atp5pb	153	0.2463
	Vdac3	169	0.2734
	Bax	193	0.2951
	Slc25a5	199	0.3204
	Ndufa4	201	0.3462
MYC TARGETS V1	Ppia	21	0.1087
	Rps3	46	0.1658
	Rps10	55	0.2248
	Rps2	71	0.2732
	Ldha	85	0.3145
	Rplp0	100	0.3542
	Cox5a	119	0.3879
	Rpl18	123	0.4240
	Rps5	126	0.4581
	Hsp90ab1	146	0.4854

Supplementary Table 3
Top GSEA Genes in MM MSC-lineage

Pathway	Gene	Gene Rank	Running ES
INTERFERON ALPHA RESPONSE	Ifitm3	1	0.1206
	Irf7	3	0.2242
	Isg15	6	0.3073
	Ly6e	7	0.3889
	Rtp4	14	0.4578
	Ifi27	15	0.5277
	Lgals3bp	18	0.5950
	B2m	33	0.6454
	Cd47	56	0.6790
	Ddx60	77	0.7023
INTEFERON GAMMA RESPONSE	Ifitm3	1	0.0990
	Irf7	3	0.1840
	Isg15	6	0.2521
	Ly6e	7	0.3190
	Xaf1	13	0.3757
	Rtp4	14	0.4332
	Ifi27	15	0.4906
	Lgals3bp	18	0.5458
	Zbp1	32	0.5879
	B2m	33	0.6315
OXIDATIVE PHOSPHORYLATION	Gpx4	46	0.3541
	Cyb5a	140	0.4554
	Vdac3	211	0.5131
	Slc25a4	247	0.5543
	Pdk4	253	0.6000
	Ldha	255	0.6455
	Cox5a	259	0.6886
	Vdac2	273	0.7270
	Oat	280	0.7652
	Slc25a5	286	0.8012

Supplementary Table 4
Top GSEA Genes in MM BMEC

Pathway	Gene	Gene Rank	Running ES
INTERFERON ALPHA RESPONSE	Bst2	0	0.1185
	Ifitm3	1	0.2161
	Irf7	2	0.2998
	Ifi44	5	0.3778
	Rtp4	9	0.4439
	Ifit3	10	0.5103
	Isg15	11	0.5765
	B2m	24	0.6178
	Rsad2	50	0.6427
	Eif2ak2	55	0.6722
INTERFERON GAMMA RESPONSE	Bst2	0	0.0953
	Ifitm3	1	0.1738
	Irf7	2	0.2411
	Ifi44	5	0.3037
	Rtp4	9	0.3567
	Ifit3	10	0.4101
	Isg15	11	0.4633
	Xaf1	12	0.5109
	B2m	24	0.5437
	Rnf213	29	0.5753
OXIDATIVE PHOSPHORYLATION	Ndufc2	131	0.0040
	Gpx4	171	0.0267
	Ldha	181	0.0569
	Etfb	194	0.0849
	Atp5h	202	0.1132
	Atp5e	210	0.1408
	Ndufa4	233	0.1620
	Cox5a	238	0.1878
	Ndufa8	251	0.2098
	Slc25a5	253	0.2346
INFLAMMATORY RESPONSE	Bst2	0	0.2499
	Irf7	2	0.4263
	Rtp4	9	0.5659
	Eif2ak2	55	0.6186
	Mmp14	97	0.6569
	Cxcl9	147	0.6831
	Chst2	188	0.7065
	Ly6e	205	0.7344
	Stab1	244	0.7522
	Calcrl	252	0.7773

Supplementary Table 5
Top ranked ligands

5TGM1-to-MSCs		5TGM1-to-OLCs		5TGM1-to-Fibroblastes		5TGM1-to-SECs		5TGM1-to-AECs		5TGM1-to-Pericytes	
ligand	rank	ligand	rank	ligand	rank	ligand	rank	ligand	rank	ligand	rank
Tgfb1	1	Tgfb1	1	Tgfb1	1	Tgfb1	1	Tgfb1	1	Tgfb1	1
Bgn	2	Cd44	2	Pf4	2	Cd44	2	Msn	2	Pf4	2
Ccn2	3	Pf4	3	Ccn2	3	Selplg	3	Col1a2	3	St6gal1	3
Pf4	4	Itgb1	4	Calr	4	Bgn	4	Ccn2	4	Saraf	4
Sema4d	5	Calm1	5	Itgb1	5	Itgb1	5	Bgn	5	H2-Q4	5.5
Itgb1	6	H2-K1	6	Bgn	6	Itga4	6	H2-K1	6	H2-Q7	5.5
Msn	7	H2-Q4	7.5	Ctsd	7	Msn	7	H2-Q4	7.5	H2-D1	7
Col1a2	8	H2-Q7	7.5	Itm2b	8	Pf4	8	H2-Q7	7.5	H2-K1	8
H2-D1	9	H2-D1	9	Lgals3bp	9	H2-K1	9	H2-D1	9	B2m	9
H2-Q4	10.5	Itga4	10	Reln	10	H2-D1	10	Calm3	10	Thbs1	10
H2-Q7	10.5	Msn	11	Itga4	11	H2-Q4	11.5	Calm1	11	Cxcl12	11
H2-K1	12	Cxcl12	12	Sema4d	12	H2-Q7	11.5	Ctsd	12	Copa	12
Lgals1	13	Lgals1	13	H2-K1	13	Arf1	13	Grn	13	Ccn2	13
Cxcl12	14	Calm2	14	H2-D1	14	Calm1	14	Pf4	14	Reln	14
Itga4	15	Col1a1	15	H2-Q4	15.5	Col1a2	15	Lgals3bp	15	Msn	15
Ppbp	16	Calm3	16	H2-Q7	15.5	Enpp1	16	Gnas	16	Hsp90b1	16
Calm1	17	Reln	17	Slpi	17	Col1a1	17	Col1a1	17	Lgals1	17
Sdc4	18	Ccn2	18	Lgals1	18	B2m	18	Enpp1	18	Col1a1	18
Enpp1	19	Sparc	19	Calm1	19	Lgals1	19	Calm2	19	Calm1	19
B2m	20	Sema4d	20	Gnas	20	Ctsd	20	B2m	20	Calm3	20
Grn	21	Sdc4	21	Msn	21	Sdc4	21	Cd79b	21	Sema4d	21
Cd47	22	Hsp90b1	22	Psap	22	Gnas	22	Lgals1	22	Calm2	22
Hsp90b1	23	Rtn4	23	Hsp90b1	23	Calm3	23	Reln	23	Calr	23
Thbs1	24	Lgals3bp	24	Calm2	24	Gpi1	24	Gpi1	24	Gpi1	24
Gpi1	25	Calr	25	B2m	25	Reln	25	Saraf	25	Cd47	25
Lgals3bp	26	Ctsd	26	Sparc	26	Calm2	26	Cd9	26	Gnas	26
Gnas	27	Col1a2	27	Enpp1	27	Grn	27	Calr	27	Sparc	27
Rtn4	28	Gnas	28	Cxcl12	28	Ccn2	28	Thbs1	28	Ctsd	28
Calm2	29	Enpp1	29	Calm3	29	Cd79b	29	Dcn	29	Lgals3bp	29
Calr	30	Copa	30	Cd79b	30	Ppbp	30	Cxcl12	30	Psap	30
Saraf	31	Grn	31	Grn	31	Cd47	31	Sparc	31	Enpp1	31
Ctsd	32	Mif	32	Col1a1	32	Thbs1	32	Slpi	32	Cd79b	32
Copa	33	Saraf	33	Gpi1	33	Lgals3bp	33	Mif	33	Grn	33
Calm3	34	Gpi1	34	Cd47	34	Entpd1	34	Alcam	34	Cd9	34
Psap	35	Psap	35	Saraf	35	Dcn	35			Col1a2	35
Reln	36	Dcn	36	Cd9	36	Copa	36			Dcn	36
Cd79b	37	Alcam	37	Dcn	37	Cd9	37			Alcam	37
Dcn	38	Thbs1	38	Mif	38	Psap	38			Mif	38

Col1a1	39	St6gal1	39	Col1a2	39	Saraf	39				
Mif	40	Slpi	40	Alcam	40	Sema4d	40				
Sparc	41			Thbs1	41	Cxcl12	41				
Cd9	42			St6gal1	42	Calr	42				
St6gal1	43			Itgal	43	Sparc	43				
Slpi	44			Adgre5	44	Slpi	44				
Epcam	45					Itgal	45				
Itgal	46					Cd24a	46				
Alcam	47					Alcam	47				
						Mif	48				
						Adgre5	49				

Supplementary Table 6

Non-stromal cells by condition

Cell Type	PBS	5TGM1
Platelets	969	1902
RBC	1185	615
B cells	7010	6453
Monocytes/macrophages	5339	1534
HSC/GMP	493	274
Cycling/proliferative	2629	1596
Neuronal cells	77	89
Total non-stromal	17702	12463
